# Compensatory mechanisms that maintain androgen production in mice lacking key androgen biosynthetic enzymes

**DOI:** 10.1096/fj.202402093R

**Published:** 2024-11-18

**Authors:** Ben M. Lawrence, Liza O'Donnell, Anne‐Louise Gannon, Sarah Smith, Michael K. Curley, Annalucia Darbey, Rosa Mackay, Peter J. O'Shaughnessy, Lee B. Smith, Diane Rebourcet

**Affiliations:** ^1^ College of Engineering, Science and Environment The University of Newcastle Callaghan New South Wales Australia; ^2^ School of BioSciences The University of Melbourne Parkville Victoria Australia; ^3^ Office of the Deputy Vice Chancellor (Research) Griffith University Southport Queensland Australia; ^4^ MRC Centre for Reproductive Health University of Edinburgh, The Queen's Medical Research Institute Edinburgh UK; ^5^ School of Biodiversity, One Health & Veterinary Medicine, College of Medical, Veterinary and Life Sciences University of Glasgow, Garscube Campus Glasgow UK; ^6^ Inserm, EHESP, Irset (Institut de recherche en santé, environnement et travail) ‐ UMR_S 1085 Univ Rennes Rennes France

**Keywords:** androgens, dihydrotestosterone, male fertility, steroids, testis, testosterone

## Abstract

Testosterone and dihydrotestosterone (DHT) are essential for male development and fertility. In the canonical androgen production pathway, testosterone is produced in the testis by HSD17B3; however, adult male *Hsd17b3* knockout (KO) mice continue to produce androgens and are fertile, indicating compensatory mechanisms exist. A second, alternate pathway produces DHT from precursors other than testosterone via 5α‐reductase (SRD5A) activity. We hypothesized that the alternate pathway contributes to androgen bioactivity in *Hsd17b3* KO mice. To investigate contributions arising from and interactions between the canonical and alternate pathways, we pharmacologically inhibited SRD5A and ablated *Srd5a1* (the predominant SRD5A in the testis) on the background of *Hsd17b3* KO mice. Mice with perturbation of either the canonical or both pathways exhibited increased LH, testicular steroidogenic enzyme expression, and normal reproductive tracts and fertility. In the circulation, alternate pathway steroids were increased in the absence of HSD17B3 but were reduced by co‐inhibition of SRD5A1. Mice with perturbations of both pathways produced normal basal levels of intratesticular testosterone, suggesting the action of other unidentified hydroxysteroid dehydrogenase(s). Strikingly, testicular expression of another SRD5A enzyme, *Srd5a2*, was markedly increased in the absence of *Hsd17b3*, suggesting a compensatory increase in SRD5A2 to maintain androgen bioactivity during HSD17B3 deficiency. Finally, we observed elevated circulating concentrations of the 11‐keto‐derivative of DHT, suggesting compensatory extra‐gonadal induction of bioactive 11‐keto androgen production. Taken together, we conclude that, in the absence of the canonical pathway of androgen production, multiple intra‐ and extra‐gonadal mechanisms cooperate to maintain testosterone and DHT production, supporting male development and fertility.

## Graphical abstract

Androgens are produced by both the canonical pathway, where HSD17B3 produces testosterone and steroid 5α‐reductase (SRD5A) converts testosterone to dihydrotestosterone (DHT), and the SRD5A‐dependent alternate pathway, where DHT is produced from other precursors. Disruption to the canonical or canonical *and* alternate pathways induces multiple compensatory mechanisms to maintain androgen production, including increased circulating LH, alternate pathway steroids and 11‐keto‐androgen, maintenance of testosterone production by other hydroxysteroid dehydrogenases, and increased testicular expression of an alternate SRD5A, *Srd5a2*, to maintain DHT production.

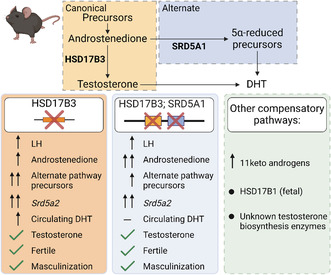

**Abbreviations**
11K: 11‐keto
11OH: 11‐hydroxy
17OH: 17‐hydroxy
17OH‐DHP: 17‐hydroxy‐5α‐dihydroprogesterone
3α‐Diol: 5α‐androstane‐ 3α, 17β‐diol
3β‐diol: 5α‐androstane‐3β, 17β‐diol
5α‐DHP: 5α‐dihydroprogesterone
AGD: anogenital distance
AKR1C: aldo‐keto reductase family 1 member C
AR: androgen receptor
CYP11A1: cytochrome P450 11A1, cholesterol side‐chain cleavage enzyme
CYP17A1: cytochrome P450 17A1
CYP19A1: cytochrome P450 family 19 subfamily A member 1
DHEA: dehydroepiandrosterone
dHet: double heterozygous
DHT: dihydrotestosterone
dKO: double knockout
hCG: human chorionic gonadotrophin
HSD17B: hydroxysteroid‐dehydrogenase‐17‐beta
HSD3B: hydroxysteroid‐dehydrogenase‐3‐beta
KO: knockout
LH: luteinizing hormone
LHCGR: luteinizing hormone/choriogonadotropin receptor
SRD5A: steroid 5α‐reductase
WT: wild‐type

## 1 INTRODUCTION

Male development, fertility, lifelong health, and well‐being are androgen‐dependent. Perturbed androgen action in men is linked with infertility/low sperm counts^1^ and an increased risk of developing chronic and age‐related conditions, including cardiovascular disease, diabetes, obesity, and metabolic syndrome.^2^, ^3^ The androgen testosterone and its 5α‐reduced derivative dihydrotestosterone (DHT) are key drivers of male sexual development and function. In males, testosterone is predominantly synthesized by Leydig cells in the testes via a single testis‐specific 17‐ketosteroid reductase enzyme, HSD17B3. Testosterone and DHT both act through the androgen receptor (AR) to promote androgen‐dependent gene transcription; however, DHT has a higher affinity for and dissociates more slowly from the AR and thus is a more potent androgen than testosterone.^4^

**FIGURE 1:**
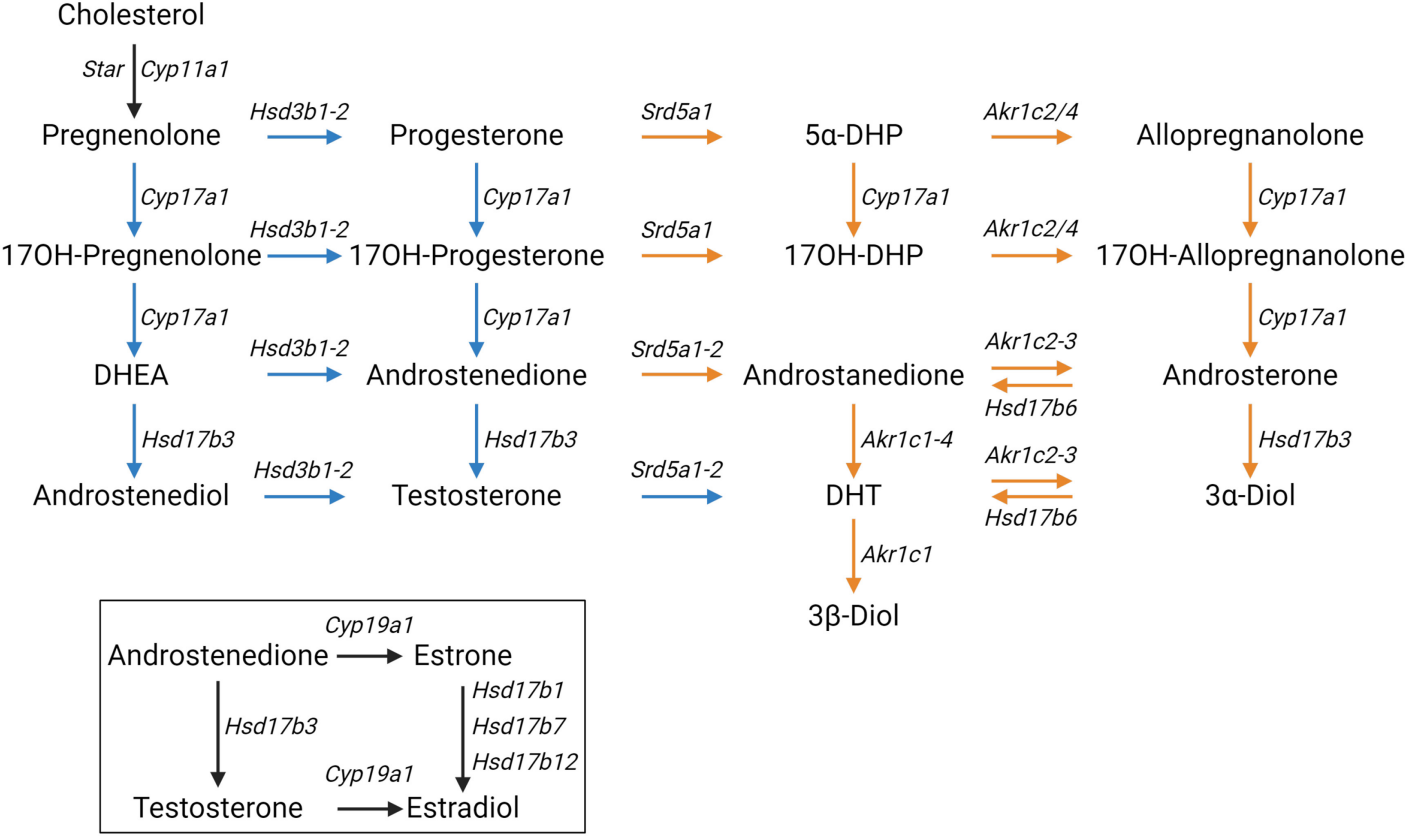
The canonical and alternate pathways of androgen biosynthesis. All androgens originate from cholesterol and are converted by multiple enzymes to produce the active androgens testosterone and dihydrotestosterone (DHT). The canonical pathway (blue arrows) produces testosterone, which can act directly on the androgen receptor or be used as a precursor to DHT. The alternate pathway (orange arrows) can synthesize DHT without the need for testosterone synthesis. The mouse gene symbols for the enzymes responsible for each reaction are shown in italics. The dashed box indicates that androstenedione and testosterone can also be converted to estrogens via CYP19A1 and are aromatized to estrone and estradiol, respectively. 17OH, 17‐hydroxy; 17OH‐DHP, 17‐hydroxy‐5α‐dihydroprogesterone; 3α‐Diol, 5α‐androstane‐3α, 17β‐diol; 3β‐Diol, 5α‐androstane‐3β, 17β‐diol; 5α‐DHP, 5α‐dihydroprogesterone; DHEA, dehydroepiandrosterone; DHT, dihydrotestosterone.

Androgen biosynthesis occurs via multiple pathways (Figure 1). The canonical pathway involves the synthesis of testosterone from androstenedione, which can then be converted to DHT by steroid 5α‐reductase (SRD5A) enzymes.^5^, ^6^, ^7^ In contrast, the alternate pathway utilizes steroid precursors other than testosterone to produce DHT.^5^, ^7^, ^8^ This pathway was first discovered in the tammar wallaby^9^, ^10^ and has since been confirmed in many species, including mice and humans.^7^, ^11^, ^12^, ^13^, ^14^ The alternate pathway entry point utilizes SRD5A to convert the precursors progesterone, 17OH‐progesterone, and androstenedione from the canonical pathway into the alternate pathway, producing 5α‐dihydroprogesterone, 17OH‐dihydroprogesterone, and androstanedione, respectively (Figure 1, ^14^). The aldo‐keto reductase family 1 member C enzymes (AKR1C1–4), the CYP17A1 enzyme, and HSD17B6 then convert alternate pathway precursors into DHT (Figure 1).

The physiological relevance of the canonical and alternate pathways to male reproductive development have been independently explored in humans.^12^, ^14^ Loss of function mutations in enzymes specific to the canonical pathway lead to disordered sexual development in humans.^12^, ^15^, ^16^, ^17^ Loss of function mutations in AKR1C2 and AKR1C4 (specific to the alternate pathway) also result in disordered sexual development due to under‐masculinization.^12^ Therefore, normal activity in both pathways appears to be essential for masculinization in human males.^12^, ^14^

Loss of function mutations to *HSD17B3* and disruption of the canonical pathway is the most common disorder of androgen synthesis, leading to disordered sexual development and male infertility.^17^ HSD17B3 deficiency is characterized by a high androstenedione to testosterone ratio^18^ due to an inability to reduce androstenedione into testosterone.^16^, ^19^, ^20^ 46,XY HSD17B3‐deficient individuals can undergo late‐onset masculinization during puberty, where male characteristics can develop.^19^, ^21^ In contrast to humans, in two independently generated *Hsd17b3* knockout (KO) mouse lines, male mice were phenotypically normal at birth, and the adults were fertile with normal levels of intratesticular testosterone^20^, ^22^ and DHT.^22^

Thus, *Hsd17b3* KO male mice maintain sufficient androgen action for sexual development and testis function. These observations point to the existence of compensatory mechanisms in mice to maintain androgen biosynthesis pathways independent of HSD17B3, and increased activity through the alternate pathway could be one such mechanism. It is also possible that 11‐keto androgens could contribute to androgen bioactivity in these mice. 11‐keto androgens are bioactive androgens synthesized from adrenal‐derived 11‐oxygenated steroids and are likely to be important in females but are present at much lower levels in males.^23^ 11‐keto androgens are the predominant bioactive androgens in fish^24^ and are present in the circulation of humans^24^, ^25^ and the testes of mice.^26^, ^27^ 11‐keto androgens have been suggested to be potent activators of the AR^24^ although in vitro they have a considerably lower potency in terms of AR activation than their native androgens.^28^ In fetal mouse testes, a decreased ability to convert androstenedione to testosterone is associated with increased 11‐keto‐testosterone levels, suggesting increased keto‐androgen biosynthesis could be a compensatory response to reduced androgen biosynthesis during fetal testis development.^27^ However, the role of 11‐keto androgens in adult male mice is not known.

The current study investigates the hypothesis that ablation of the canonical pathway in mice induces compensatory mechanisms to maintain androgen bioactivity. We hypothesized that the canonical and alternate pathways cooperate to maintain male sexual development and adult testis function and fertility in mice. To investigate this, we created mice lacking both HSD17B3 and SRD5A1. SRD5A1 is the predominant SRD5A in the rodent pubertal and adult testis^13^, ^29^ and is likely, therefore, to be an essential gateway entry point into the alternate pathway (Figure 1). We examined whether SRD5A1 and the alternate pathway of androgen biosynthesis maintain DHT production and preserve testis development and fertility in *Hsd17b3* KO male mice.

## 2 MATERIALS AND METHODS

### 2.1 Transgenic mice

The *Hsd17b3* KO mice used for the dutasteride study were generated as previously described,^20^ and experiments were performed at the University of Edinburgh under the UK Animal Scientific Procedures Act, Home Office License number PPL 70/8804.

Both *Hsd17b3* and *Srd5a1* are expressed as early as embryonic day 13.5 in the fetal mouse testis.^26^ *Hsd17b3 and Srd5a1* double knockout (dKO) mice were generated by the MEGA Genome Engineering Facility at the Garvin Institute of Medical Research, Darlinghurst, NSW. Crispr/Cas9 was used to generate a 7‐base pair deletion at the end of exon 1 in both genes, which caused a frameshift mutation. For general colony management, *Hsd17b3* ^*+/−*^; *Srd5a1* ^*+/−*^ males and females were bred together. Due to the low percentage of recombination occurring between the *Hsd17b3* and *Srd5a1* genes on chromosome 13, mice with a mixed genotype were used to generate more of that specific genotype. The following genotypes were used in this study: *Hsd17b3* ^+/+^; *Srd5a1* ^+/+^ (wild type, WT), *Hsd17b3* ^+/−^; *Srd5a1* ^+/+^ (*Hsd17b3* Het, *Srd5a1* WT), *Hsd17b3* ^+/−^; *Srd5a1* ^+/−^ (double heterozygous, dHet), *Hsd17b3* ^−/−^; *Srd5a1* ^+/−^ (*Hsd17b3* KO), and *Hsd17b3* ^−/−^; *Srd5a1* ^−/−^ (double KO, dKO). Mice were exposed to a 12‐h day/night cycle and had access to soy‐free chow to prevent potential estrogenic effects and to fresh drinking water ad libitum. All procedures were approved by the University of Newcastle's Animal Care and Ethics Committee (ACEC; approval #A‐2018‐820). All animal experiments were performed in accordance with the Australian code of practice for the care and use of animals for scientific purposes by the National Health and Medical Research Council of Australia.

### 2.2 In vivo treatments

Dutasteride (Sigma‐Aldrich, Gillingham, United Kingdom) was used to inhibit 5α‐reductase enzymes. Wild type, heterozygous (together referred to as controls), and *Hsd17b3* KO mice were treated daily with either a vehicle or 1.8 mg/kg/d dutasteride^30^ in their diet from day 50, and tissues were collected 30 days later. Adult mice received a single 20 IU intraperitoneal injection of human chorionic gonadotrophin (hCG) (Sigma‐Aldrich, Australia) as previously described.^20^ Tissues/serum were collected 16 h post injection.

### 2.3 Tissue collection

Adult mice were killed by inhalation of CO_2_, whereas day 0 mice were killed by decapitation. Blood was collected by cardiac puncture and serum obtained by centrifugation at 4°C for 10 min and then snap frozen and stored at −80°C. Anogenital distance (AGD) was measured using digital calipers. Tissues were collected aseptically, weighed, and either fixed in Bouin's solution (2 h for neonatal tissues, 6 h for adult tissues) for histological analysis or snap frozen and stored at −80°C for RNA, sperm quantification, or steroid analysis.

### 2.4 Genotyping

Genotyping was performed on ear biopsies after weaning and a second time on tail clips to verify the genotype postmortem. Genomic DNA (gDNA) was digested in Tris‐EDTA‐Tween, pH 8, and Proteinase K (20 μg per ear biopsy or 40 μg per tail clip) for 1 h at 55°C, followed by 7 min at 95°C to denature remaining Proteinase K. Digested samples were diluted 1:10 in DEPC‐treated DNase‐ and RNase‐free sterile water.

The genotype of transgenic mice was identified by transgene‐specific PCR assays. PCR was performed on gDNA extracts using a Type‐it Mutation Detect PCR Kit (QIAGEN, VIC, Australia). Details of genotyping PCR assays are included in Supplemental Information (Tables S1 and S2). PCR products were analyzed on the QIAxcel Advanced System using QIAxcel ScreenGel Software (QIAGEN, VIC, Australia). The amplified DNA was detected by a QIAxcel DNA high resolution kit (QIAGEN, VIC, Australia), and PCR product sizes were recorded.

### 2.5 Epididymal sperm reserve evaluation

Epididymal sperm were quantified from frozen epididymal samples. Epididymides were thawed on ice, and the cauda epididymis was removed and homogenized in 0.9% NaCl and 0.5% Triton X‐100 using an Eppendorf micropestle (Eppendorf, Macquarie Park, NSW, Australia). Elongated spermatids were counted in a Neubauer hemocytometer cell counting chamber (Adelab Scientific, Thebarton, SA) by a blinded observer.

### 2.6 Quantitative RT‐PCR

RNA was extracted using the RNeasy Mini kit (QIAGEN, VIC, Australia) as per manufacturer's instructions, including RNase‐free DNase on‐column digestion (QIAGEN, VIC, Australia). For RNA extraction of adult mouse tissue, an external Luciferase RNA control (Promega, Alexandria, NSW, Australia) was added at 1 ng/20 mg tissue to the homogenized tissue. RNA concentration was measured using a NanoDrop Lite Spectrophotometer (Thermo Fisher Scientific, VIC, Australia). Extracted RNA was reverse transcribed to synthesize cDNA using the SuperScipt VILO cDNA Synthesis Kit (Thermo Fisher Scientific, VIC, Australia) as per manufacturer's instructions. A reverse transcriptase negative (−RT) control and a water (no template) control were included in all reverse transcriptions.

For qRT‐PCR, target‐specific primers and corresponding specific probes were identified and selected using the online Roche Universal Probe Library (UPL) Assay Design Centre. qRT‐PCR was performed on the LightCycler 96 system (Millenium Science, Mulgrave, VIC, Australia). Concentrations of reagents and details of primers and UPL probes are listed in Supplementary Information (Tables S3 and S4). Quantification of mRNA expression was calculated using the 2^−ΔΔCt^ method. Gene expression was determined relative to *Beta‐actin* in neonatal tissues (Universal ProbeLibrary mouse *Beta‐actin* gene assay, Sigma‐Aldrich, Australia) and relative to the external housekeeping Luciferase gene in adult tissues (Roche, AU). As each sample was run in triplicate, an average of the Ct value was taken.

### 2.7 Hormone analysis

Serum LH levels in mice were measured using a commercially validated sandwich LH (Rodent) enzyme‐linked immunosorbent assay (ELISA) kit, #KA2332 (Abnova, Taiwan). Steroids were quantified in serum and testis homogenates from mice to determine circulating and intratesticular hormone levels, respectively. Samples were thawed and kept cold on ice prior to analysis. Fragments of adult testes (20–40 mg) and whole neonatal testes were weighed and homogenized in 50 mM Tris pH 7.4, 1% deoxycholate, 0.01% SDS, containing PhosSTOP (Sigma‐Aldrich, Australia) and cOmplete Mini Protease Inhibitor Cocktail (Sigma‐Aldrich, Australia) at a concentration of 20 μL/mg of adult testis tissue or 100 μL/mg for neonatal testis tissue. Samples were homogenized for 4 × 30 s increments at 25 Hz, with 1 min on ice between each interval to ensure samples did not overheat. Samples were stored at −80°C until analysis. Mass spectrometry steroid analysis was performed at the ANZAC Research Institute in Concord West, NSW. Details of steroids analyzed and detection limits are listed in Supplemental Information (Table S5).

### 2.8 Tissue histology

Fixed tissues were processed, paraffin‐embedded, and 5 μm sections were prepared. Sections were dewaxed in xylene (Sigma‐Aldrich, Australia) and rehydrated through a series of decreasing ethanol gradients. Tissue sections were stained with hematoxylin and eosin (Sigma‐Aldrich, Australia).

### 2.9 Microscopy

Testis and epididymis of day 0 newborn neonates were imaged using a ZEISS SteREO Discovery.V12 Microscope (Carl Zeiss AG, Germany). Light microscopy images of adult tissue sections were captured using a Zeiss AXIO Imager A1 microscope (Carl Zeiss AG, Germany). Images were accessed through ZEN imaging software (Carl Zeiss AG, Germany).

### 2.10 Statistical Analysis

Statistical analyses were performed using GraphPad Prism 8.4.3 software (GraphPad Software, San Diego, CA, USA). The Gaussian distribution of datasets was assessed by the Shapiro–Wilk normality test to determine if parametric or nonparametric statistical testing would be most appropriate. Datasets that passed the normality test underwent parametric tests, including one‐way ANOVA with Tukey's post hoc test and two‐way ANOVA with Tukey's post hoc test when two independent variables were present. Nonparametric statistical testing was applied to datasets that did not pass the normality test, and statistical significance was determined using a Kruskal–Wallis test and Dunn's multiple comparisons posthoc analysis. Data were considered significantly different when the p‐value was ≤0.05. Data are presented as the mean with the standard error of the mean (SEM). Power calculations were performed by GraphPad StatMateTM 2.00 software to identify an appropriate group size required for hormone analysis.

## 3 RESULTS

### 3.1 Dutasteride, an inhibitor of 5α‐reductase enzyme activity, does not alter testis size or morphology in adult male mice lacking HSD17B3

To establish whether SRD5A and the alternate pathway of androgen biosynthesis contribute to the preserved androgen action in adult *Hsd17b3* KO mice, we first used a well‐established pharmacological inhibitor of SRD5A activity, dutasteride, which is a competitive inhibitor of both SRD5A type 1 and 2 enzymes (SRD5A1 and SRD5A2). Wild‐type and *Hsd17b3* KO mice (50 days old) were exposed to either dutasteride or vehicle treatment in their diet for 30 days, with tissue collected at day 80.

**FIGURE 2:**
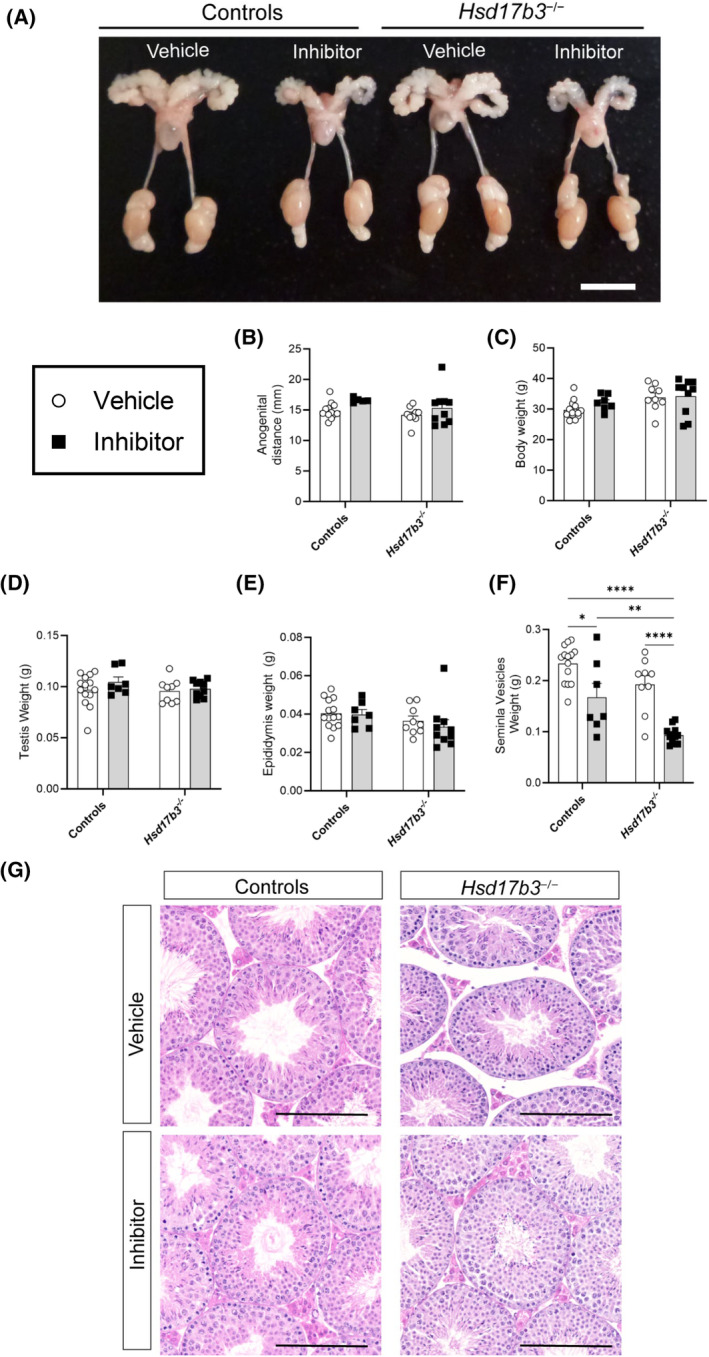
Competitive inhibition of 5α‐reductase enzymes (SRD5A1 and SRD5A2) by dutasteride. (A) Representative images of the male reproductive tract of adult control and *Hsd17b3* ^−/−^ (*Hsd17b3* knockout [KO]) mice following 30 days of vehicle or dutasteride (inhibitor) treatment. Scale bar: 10 mm. (B) Anogenital distance and (C) total body weight of controls and *Hsd17b3* KO post‐treatment of vehicle or inhibitor. Reproductive tissue weights of the (D) testis, (E) epididymis, and (F) seminal vesicles in control and *Hsd17b3* KO mice post vehicle or inhibitor treatment. Two‐way ANOVA, Tukey's test, where *p* ≤ .05, data shown as mean ± SEM with *n* = 7–14 per group. Significant differences between groups are indicated as *p ≤ .05, **p ≤ .01, ****p ≤ .0001. (G) Representative images of hematoxylin and eosin (H&E) staining of controls and *Hsd17b3* ^−/−^ (*Hsd17b3* knockout) mouse testes after vehicle or dutasteride (inhibitor) treatment. Scale bar: 200 μm.

Seminal vesicle weight is highly dependent on DHT, and a reduction in seminal vesicle weight is observed after the administration of SRD5A inhibitors^31^, ^32^; thus, we assessed impacts on this organ. Dutasteride caused a significant reduction in seminal vesicle weight in both wild‐type and *Hsd17b3* KO mice (Figure 2F); however, anogenital distance (AGD) did not change, and there were no changes in body, testis, or epididymis weights after dutasteride treatment (Figure 2B–E). The gross morphology of the testis of wild‐type and *Hsd17b3* KO mice was also unaffected by dutasteride treatment (Figure 2G). These results suggest that SRD5A inhibition in adult *Hsd17b3* KO mice does not affect testis weight or gross histology.

### 3.2 Circulating steroid analysis in dutasteride‐treated mice reveals that the canonical and alternate pathways of androgen biosynthesis co‐operate to maintain androgen production

**FIGURE 3:**
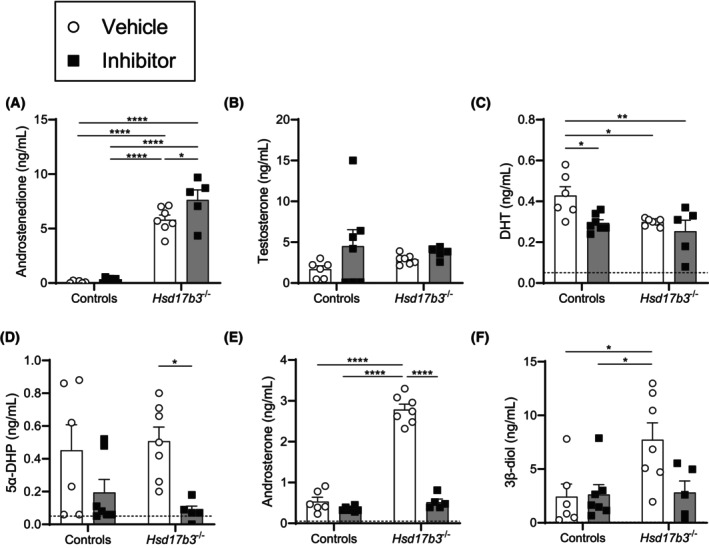
Alternate pathway androgen precursors were elevated in mouse serum in the absence of *Hsd17b3* yet were abrogated by the addition of a 5α‐reductase inhibitor. Control or *Hsd17b3* ^−/−^ (*Hsd17b3* knockout [KO]) mice were treated with either vehicle or dutasteride (5α‐reductase inhibitor) in the diet for 30 days and were collected on day 80. (A) Steroids assessed in serum include androstenedione, (B) testosterone, (C) dihydrotestosterone (DHT), (D) 5α‐dihydroprogesterone (5α‐DHP), (E) androsterone, and (F) 5α‐androstane‐*3β*,17β‐diol (3β‐diol). Biological replicates that were below the limit of detection were recorded as 0 ng/mL. The limit of detection ranged from 0.01 ng/mL to 0.05 ng/mL depending on the analyte and is indicated by a dotted black line on the *y*‐axis. Two‐way ANOVA, Tukey's test where *p* ≤ .05, data shown as mean ± SEM with individual values for *n* = 5–7 biological replicates per group. Significant differences between groups are indicated as *p ≤ .05, **p ≤ .01, ****p ≤ .0001.

Key steroids from the canonical and alternate androgen pathways were quantified in the circulation of mice treated with dutasteride. Androstenedione levels were significantly increased in *Hsd17b3* KO mice compared to controls (Figure 3A), due to a reduced ability to convert androstenedione to testosterone as previously described.^15^, ^18^ Dutasteride treatment caused a further increase in androstenedione levels in *Hsd17b3* KO mice compared to vehicle‐treated *Hsd17b3* KO mice (Figure 3A), suggesting an accumulation of substrate steroids due to the reduced ability for these to be converted to other steroid products via the alternate pathway. Testosterone levels were unchanged across the different groups (Figure 3B). DHT levels in circulation were significantly decreased in dutasteride‐treated control mice compared to vehicle‐treated controls (Figure 3C). DHT levels were also reduced in vehicle‐treated and dutasteride‐treated *Hsd17b3* KO mice compared to vehicle‐treated controls; however, no significant difference was observed between these two groups (Figure 3C). Taken together, these results demonstrate that dutasteride in control mice reduced serum DHT to a level similar to *Hsd17b3* KO mice; however, dutasteride was unable to further reduce DHT in *Hsd17b3* KO mice, suggesting the observed reductions in DHT arising from treatment or genotype were not additive.

To investigate the impact of dutasteride on the alternate pathway of androgen biosynthesis, precursor androgens in the alternate pathway, including 5α‐DHP, androsterone, and 5α‐androstane‐3β, 17β‐diol (androstanediol, or 3β‐diol), were investigated (Figure 3D–F). Androsterone and androstanediol (3β‐diol) were significantly increased in vehicle‐treated *Hsd17b3* KO mice compared to control mice (Figure 3E,F), suggesting that blockade of the HSD17B3‐dependent canonical pathway increased the synthesis of alternate pathway steroids. Importantly, dutasteride‐treatment in *Hsd17b3* KO mice significantly decreased 5α‐DHP and androsterone compared to vehicle‐treated KO mice (Figure 3D,E), indicating that suppression of SRD5A by dutasteride can decrease the alternate pathway of androgen biosynthesis in *Hsd17b3* KO mice.

These results suggest that the canonical and alternate pathways of androgen biosynthesis cooperate to maintain androgen bioactivity in adult male mice, however whether this is also the case during pre‐ and postnatal development is unknown.

### 3.3 Development and validation of a *Hsd17b3* and *Srd5a1* double knockout mouse model

To investigate the contribution of SRD5A and the alternate pathway of androgen biosynthesis in *Hsd17b3* KO mice during development, where dutasteride treatment presents significant challenges, a double KO mouse model of both *Hsd17b3* and *Srd5a1* was generated. SRD5A1 was chosen because it is the predominant SRD5A operating in the testis and a gateway enzyme into the alternate pathway^7^, ^21^ (Figure 1).

*Hsd17b3* and *Srd5a1* genes are in close proximity on chromosome 13 in the mouse, with *Hsd17b3* located at site 33.26 cM and *Srd5a1* at site 35.55 cM, as per the National Center for Biotechnology Information [NCBI], thus separated on the chromosome by just 2.29 cM (Figure S1A). Due to this proximity, it was rare for a recombination to occur between the two genes, making it impractical to generate double KOs from cross‐breeding individual KOs. Instead, Crispr/Cas technology was used to simultaneously generate independent 7 bp deletions at the end of exon 1 in both genes to trigger frameshift mutations (Figure S1B).

Conversely, once generated, the reduced frequency of recombination between targeted alleles of the two genes presented challenges in collecting all possible genotypes at numbers sufficient to power downstream studies. For studies in adult mice, we were able to collect the appropriate numbers of the informative genotypes of wild type (*Hsd17b3* ^*+/+*^; *Srd5a1* ^*+/+*^), double heterozygous (dHet *Hsd17b3* ^*+/−*^; *Srd5a1* ^*+/−*^), *Hsd17b3* KO (*Hsd17b3* ^*−/−*^; *Srd5a1* ^*+/−*^) and double KO (dKO, *Hsd17b3* ^*−/−*^; *Srd5a1* ^*−/−*^). Studies in newborn mice required retrospective genotyping, and, by chance, no *Hsd17b3* KO males were collected from this cohort; thus, we were only able to collect appropriate numbers of wild‐type, dHet, and dKO mice. We have thus restricted our analyses and conclusions to those that can reliably be made from the available data.

However, a previous study has shown that *Hsd17b3* KO have normal AGD and normal levels of testicular androstenedione and testosterone at birth.^33^ Also, it has previously been demonstrated that male mice heterozygous for the *Hsd17b3* knockout alleles are indistinguishable from wild types^20^ and that male mice heterozygous for the *Srd5a1* knockout allele are able to breed normally.^34^ In our study, mice that had a recombination resulting in a *Srd5a1* single KO were all female at birth. As *Srd5a1* KO in female mice results in a parturition defect,^35^ these mice were unable to be used to generate male *Srd5a1* single KO offspring. However, it has previously been shown that male homozygous *Srd5a1* KO mice develop normally and sire normal numbers of offspring.^35^

The genotypes were identified from ear or tail DNA using standard PCR (Figure S1C). Successful disruption to both pathways was confirmed by hormone analysis. Ablation of HSD17B3 was confirmed by a low intratesticular testosterone to androstenedione ratio in *Hsd17b3* KO and dKO mice (Figure S1D). *Hsd17b3* KO mice also exhibited a significantly higher ratio of circulating androsterone to androstenedione compared to WT, *Hsd17b3* ^+/−^; *Srd5a1* ^+/+^, and dHet controls (Figure S1E), indicative of increased alternate pathway steroids in the absence of *Hsd17b3*, consistent with observations in the dutasteride study (Figure 3E,F). The decreased ratio of circulating androsterone to androstenedione in dKO compared to *Hsd17b3* KO mice (Figure S1E) is consistent with decreased SRD5A activity and a decrease in the alternate pathway of androgen biosynthesis. Together, these data confirm the successful production of a mouse model lacking both HSD17B3 and SRD5A1.

### 3.4 Fetal development of male mice is not impacted by the loss of HSD17B3 and SRD5A1

Loss of function mutations in alternate pathway enzymes result in disorders of sexual development in humans^12^, ^15^, ^36^; however, whether the alternate pathway has a role during mouse fetal development is not clear. We therefore investigated the impact of manipulating both the canonical and alternate pathways of androgen biosynthesis on fetal development of the male reproductive tract. Neonatal pups were collected on the day of birth (day 0). As mentioned above, *Hsd17b3* KO mice at day 0 have normal levels of testicular androstenedione and testosterone and a normal AGD, due to the ability of HSD17B1 to compensate for the lack of HSD17B3 during fetal development.^33^

There were no gross changes in the phenotype of the dKO testes or epididymides compared to WT or dHet mice at day 0 (Figure S2A). Epididymal coiling, known to be androgen‐dependent, was noted in all genotypes. The cell composition of the day 0 testis was normal, with seminiferous tubule and interstitial cell histology similar across all genotypes (Figure S2D). No significant differences were detected in male body weight (Figure S2C), nor in male or female AGD (Figure S2B). Previous studies have shown that AGD in neonatal male mice lacking *Hsd17b3* is also normal; however, the AGD of male mice lacking both *Hsd17b1* and *Hsd17b3* is similar to females,^33^ highlighting that HSD17B1 and HSD17B3 can compensate for each other during fetal development.^33^

Thus, there were no observable gross differences in the sexual development of dKO males compared to controls, pointing to the existence of compensatory mechanisms to maintain androgen production during fetal life.

### 3.5 Androgen biosynthesis in neonatal *Hsd17b3* and *Srd5a1* double knockout mice indicates the existence of compensatory mechanisms to maintain the canonical and alternate pathways

**FIGURE 4:**
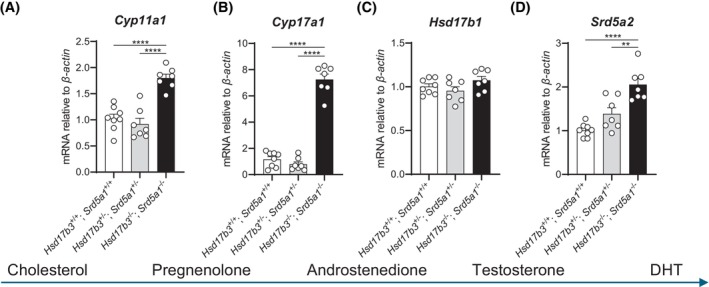
Analysis of testicular mRNA expression of steroidogenic enzymes involved in androgen biosynthesis in the neonatal *Hsd17b3* ^−/−^ and *Srd5a1* ^−/−^ (double knockout, dKO) mouse testis (day 0). (A) mRNA transcript levels of cholesterol side‐chain cleavage enzyme (*Cyp11a1*), (B) cytochrome P450 family 17 subfamily A member (*Cyp17a1*), (C) 17β‑hydroxysteroid dehydrogenase type 1 (*Hsd17b1*), and (D) steroid 5α‐reductase type 2 (*Srd5a2*) in the testis of mice on the day of birth (day 0). One‐way ANOVA, Tukey's test where *p* ≤ .05, data shown as mean ± SEM with *n* = 7–8 per group. Significant differences between groups are indicated as **p ≤ .01, ****p ≤ .0001. mRNA transcripts of enzymes (A–D) are shown according to the steroids produced at certain steps of the androgen production pathway (blue arrow).

We next investigated the effects of combined deletion of *Hsd17b3* and *Srd5a1* on androgen biosynthesis at birth (day 0). First, we assessed the testicular expression of key enzymes involved in androgen production (Figure 4). The expression of the cholesterol side‐chain cleavage enzyme cytochrome P450 family 11 subfamily A member (*Cyp11a1*) and the enzyme cytochrome P450 family 17 subfamily A member (*Cyp17a1*), both specifically expressed in Leydig cells, were significantly increased in the testis of dKO mice compared to WT and dHet animals (Figure 4A,B). The expression of *Hsd17b1*, an enzyme that has previously been shown to contribute to testosterone production during mouse fetal development,^33^ was unchanged in the dKO testis (Figure 4C). Importantly, the expression of the type 2 5α‐reductase enzyme, *Srd5a2*, was significantly increased in the dKO mouse testis (Figure 4D), suggesting that increased SRD5A2 could be a compensatory response to the loss of *Hsd17b3* and *Srd5a1* in the fetal testis.

**FIGURE 5:**
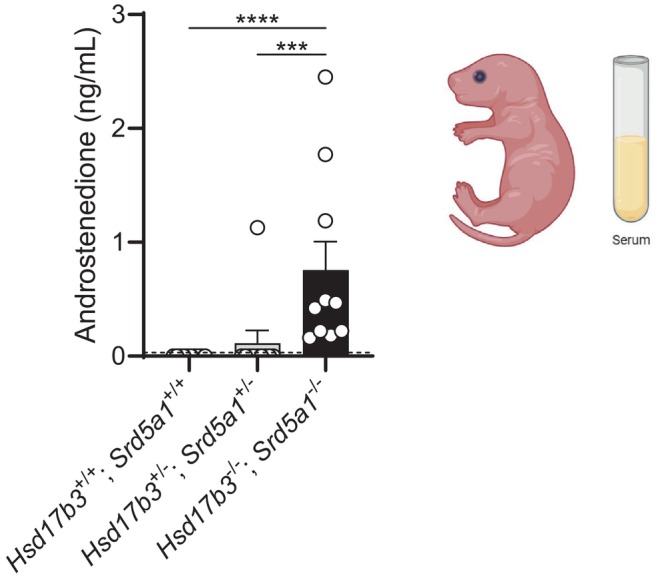
Circulating levels of androstenedione are elevated in neonatal *Hsd17b3* ^−/−^ and *Srd5a1* ^−/−^ double knockout (dKO) mice. Androstenedione levels in the serum of *Hsd17b3* ^+/+^; *Srd5a1* ^+/+^ (wild type), *Hsd17b3* ^+/−^; *Srd5a1* ^+/−^ (double heterozygous), and *Hsd17b3* ^−/−^; *Srd5a1* ^−/−^ (dKO) males on the day of birth. Samples where androstenedione was below the limit of detection were recorded as 0 ng/mL. Limit of detection: 0.03 ng/mL, indicated by dotted black line. One‐way ANOVA, Kruskal–Wallis test where *p* ≤ .05, data shown as mean ± SEM with *n* = 10 per group. Significant differences between groups are indicated as ***p ≤ .001, ****p ≤ .0001.

Circulating steroids in day 0 neonatal pups were measured by mass spectrometry (Figure 5). Androstenedione was the only analyte to be detected consistently in any cohort, with the other steroids, including testosterone, androsterone, and DHT, below the limits of detection. Circulating androstenedione levels were predominantly undetectable in WT and dHet mice but were detected in all dKO mice (Figure 5). This data suggests that neonatal dKO mice have significantly increased androstenedione levels compared to WT and dHet animals due to an inability to efficiently convert androstenedione into testosterone or other androgen precursors, as previously observed in adult *Hsd17b3* KO mice.^20^

**FIGURE 6:**
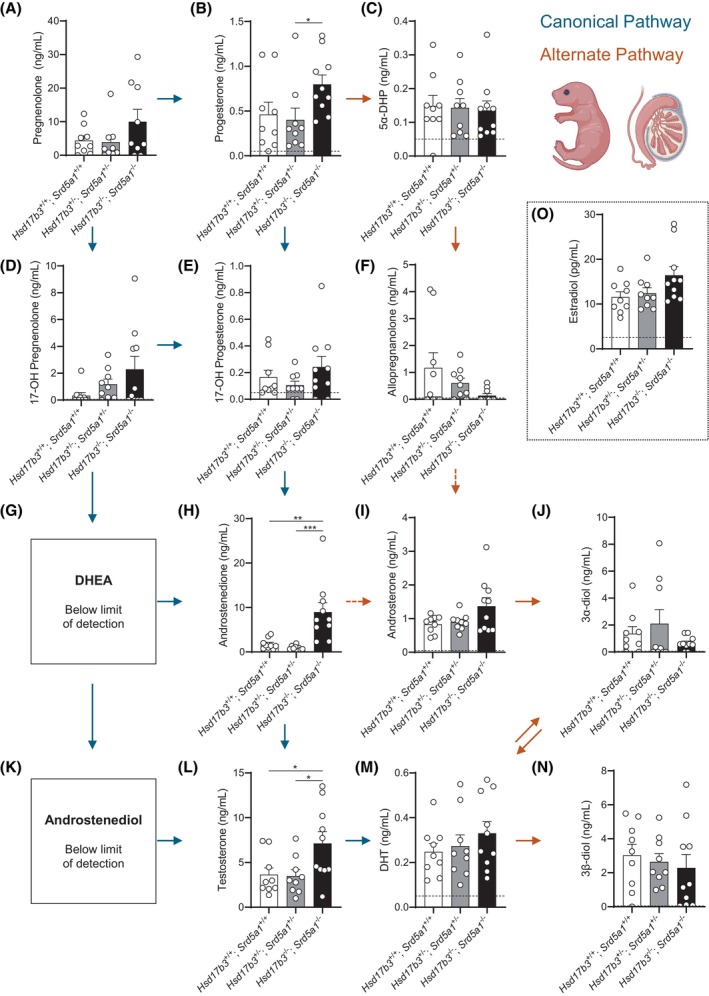
Analysis of intratesticular steroids in the mouse testis on the day of birth. Steroids were measured in testes from *Hsd17b3* ^+/+^; *Srd5a1* ^+/+^ (wild type), *Hsd17b3* ^+/−^; *Srd5a1* ^+/−^ (double heterozygous), and *Hsd17b3* ^−/−^ and *Srd5a1* ^−/−^ (double knockout, dKO) mice. (A–M) Quantitation of intratesticular androgen precursors and active androgens involved in the canonical and alternate androgen production pathways. Steroids measured include (A) pregnenolone, (B) progesterone, (C) 5α‐dihydroprogesterone (5α‐DHP), (D) 17‐OH pregnenolone, (E) 17‐OH progesterone, (F) allopregnanolone, (G) dehydroepiandrosterone (DHEA), (H) androstenedione, (I) androsterone, (J) 5α‐androstane‐3α, 17β‐diol (3α‐diol), (K) androstenediol, (L) testosterone, (M) dihydrotestosterone (DHT), (N) 5α‐androstane‐3β, 17β‐diol (3β‐diol) and (O) estradiol. Blue arrows indicate the direction of the canonical androgen production pathway. Orange arrows indicate conversion occurring in the alternate androgen production pathway. Dotted arrows indicate an indirect conversion. Biological replicates that were below the limit of detection were recorded as 0 ng/mL. The limit of detection ranged from 2.5 pg/mL to 0.05 ng/mL depending on the analyte and is indicated by a dotted black line. One‐way ANOVA, Tukey's test (for parametric data) or Kruskal–Wallis test (for nonparametric data), where *p* ≤ .05, data shown as mean ± SEM with *n* = 9–10 per group. Significant differences between groups are indicated as *p ≤ .05, **p ≤ .01, ***p ≤ .001.

Next, we measured intratesticular steroid levels in neonatal day 0 mice by mass spectrometry to assess steroidogenesis in the context of disruption of both the canonical and alternate pathways (Figure 6). No changes were observed between any genotype in pregnenolone, 17‐OH pregnenolone, or 17‐OH progesterone, which are all precursors in the canonical androgen pathway (Figure 6A,D,E), however, there was a significant increase in progesterone levels in the dKO testis compared to dHet mice (Figure 6B). DHEA and androstenediol, which are synthesized via the Δ5 route of the canonical pathway, were below the limit of detection (Figure 6G,K) likely because mice favor the Δ4 route, where *Cyp17a1* can more efficiently convert 17‐OH progesterone into androstenedione as opposed to converting 17‐OH pregnenolone into DHEA.

Intratesticular androstenedione was significantly increased in dKO mice compared to WT and dHet animals (Figure 6H); however, no changes were previously observed in neonatal *Hsd17b3* KO mice.^33^ These observations suggest an accumulation of precursor steroids due to the inability of the dKO testes to efficiently convert androstenedione via the canonical and alternate pathways. Previous studies have shown that intratesticular testosterone levels were normal in *Hsd17b3* KO mice at day 0^33^; however, in neonatal dKO mice, intratesticular testosterone levels were significantly increased compared to WT and dHet animals (Figure 6L). Taken together, these observations suggest that, in neonatal dKO mice, elevated levels of androstenedione precursor are due to the deletion of both *Hsd17b3* and *Srd5a1* preventing androstenedione metabolism via the canonical and alternate pathways, and that testosterone continues to be converted from androstenedione via neonatal expression of HSD17B1 (Figure 4C, ^33^) and potentially other, as yet unidentified, hydroxysteroid dehydrogenases.^20^, ^33^

There were no significant differences in alternate pathway androgen precursors including 5α‐DHP, allopregnanolone, androsterone, 5α‐androstane‐3α,17β‐diol (3α‐diol), and 5α‐androstane‐3β,17β‐diol (3β‐diol) between any genotype collected (Figure 6C,F,I,J,N). Similarly, intratesticular DHT levels were unaffected in dKO mice (Figure 6M). These data suggest that the alternate androgen production pathway is maintained at birth in the testes of dKO mice, likely due to testicular expression of *Srd5a2* (Figure 4D).

Finally, we investigated estrogenic steroids that are produced via the aromatase enzyme (CYP19A1) and can be converted via androstenedione or testosterone into the weak estrogen, estrone (E1), and the potent estrogen, estradiol (E2), respectively (Figure 1). Whilst estrone levels were below the limit of detection (data not shown), intratesticular estradiol was detected in all samples but showed no significant changes between WT, dHet, and dKO mice (Figure 6O). This data suggests that the disruption to the canonical and alternate androgen pathways of biosynthesis does not impact estrogen production at birth.

Taken together, the above data suggest that, when *Hsd17b3* and *Srd5a1* are deleted during fetal development, the canonical and alternate pathways of androgen biosynthesis in the testis are preserved via compensatory action of other enzymes, including HSD17B1 and SRD5A2.

### 3.6 The ablation of *Hsd17b3* and *Srd5a1* does not impact the postnatal development of the adult male reproductive system

To examine the postnatal impact of the loss of *Hsd17b3* and *Srd5a1* on adult male mice, the gross morphology of the reproductive system in adult (day 80) dKO animals was analyzed. These mice were compared to mice lacking *Hsd17b3* alone. The results showed that male reproductive organs were grossly normal, including the seminal vesicles, prostate, vas deferens, epididymides, and testes in both adult *Hsd17b3* KO and dKO (Figure S3A). Although the AGD was not changed in dKO mice at birth (Figure S2B), it was decreased in adult *Hsd17b3* KO and dKO mice compared to all other groups (Figure S3B), suggesting perturbed androgen action during postnatal development such that the prenatally‐programmed maximum AGD could not be reached.^37^ The AGD of dKO mice was not reduced compared to *Hsd17b3* KO mice, indicating that the removal of *Srd5a1* does not further influence the AGD (Figure S3B). Body weights were consistent amongst all genotypes with the exception of *Hsd17b3* KO mice, which showed a slight but significant decrease compared to WT controls and dKO mice (Figure S3C); however, this has not previously been observed in the *Hsd17b3* single KO studies.^20^, ^22^ There were no differences in testis, epididymis, or seminal vesicle weights between genotypes, which are biomarkers of intratesticular and circulating androgens (Figure S3D–F). A reduction in kidney weight was observed in *Hsd17b3* KO mice compared to WT animals, however no other changes were seen among any other genotype (Figure S3G). Finally, the weight of gonadal fat in dKO mice was increased compared to dHet animals only, suggesting possible changes in peripheral testosterone or estrogen levels (Figure S3H). As no difference was observed in any of these endpoints between *Hsd17b3* ^+/+^; *Srd5a1* ^+/+^ and *Hsd17b3* ^+/−^; *Srd5a1* ^+/+^ mice, these groups were combined for all further analyses on adult mice and are referred to as “controls.”

### 3.7 Ablation of *Hsd17b3* and *Srd5a1* does not alter fertility in adult male mice

**FIGURE 7:**
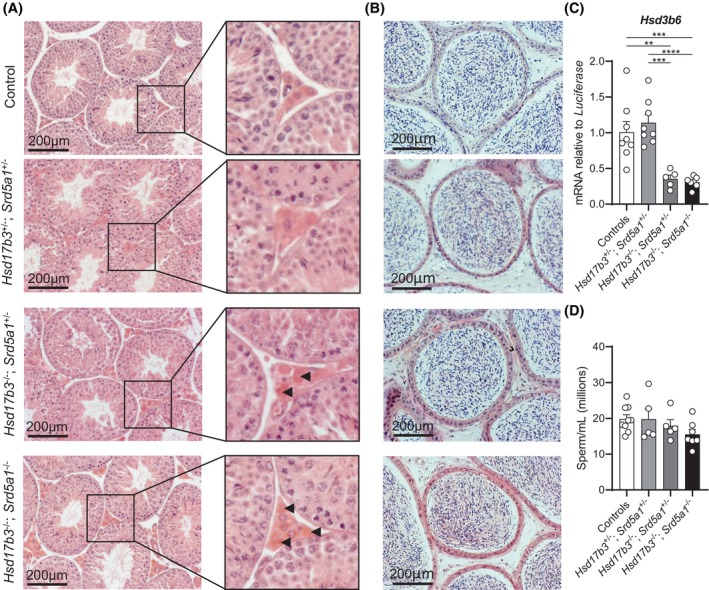
Normal sperm production in adult *Hsd17b3* and *Srd5a1* double knockout (dKO) mice. (A) Hematoxylin and eosin (H&E) staining of day 80 adult testis from control (*Hsd17b3* ^+/+^; *Srd5a1* ^+/+^ [wild type]), *Hsd17b3* ^+/−^; *Srd5a1* ^+/−^ (double heterozygous [dHet]), *Hsd17b3* ^−/−^; *Srd5a1* ^+/−^ (*Hsd17b3* knockout [KO]) and *Hsd17b3* ^−/−^; *Srd5a1* ^−/−^ (dKO) mice. The black box indicates the further magnified section of the image. Black arrowheads indicate Leydig cells. Scale bar: 200 μm. (B) Representative H&E staining images of day 80 adult cauda epididymis from control, dHet, *Hsd17b3* KO, and dKO mice. Scale bar: 200 μm. (C) mRNA transcript levels relative to a luciferase external control of 3β‐hydroxysteroid dehydrogenase type 6 (*Hsd3b6*) in adult testis tissue. One‐way ANOVA, Tukey's test where *p* ≤ .05, data shown as mean ± SEM with *n* = 5–8 per group. Significant differences between groups are indicated as **p ≤ .01, ***p ≤ .001, ****p ≤ .0001. (D) Epididymal sperm counts from the cauda epididymis. One‐way ANOVA, Tukey's test where *p* ≤ .05, data shown as mean ± SEM with *n* = 5–9 per group.

In the testis, the gross morphology of the seminiferous tubules and interstitial cells was normal across all groups (Figure 7A). Relative quantification of the mRNA transcript levels of *Hsd3b6*, a Leydig cell maturation marker, revealed a significant decrease in *Hsd17b3* KO and dKO mice compared to controls and dHet mice (Figure 7C). The cauda epididymis had grossly normal morphology, and the lumen contained mature sperm (Figure 7B). There were no significant differences in the number of epididymal sperm (Figure 7D). Fertility in adult dKO male mice appeared normal, as dKO male mice were able to sire litters (*n* = 5 mice). dKO males sired normal‐sized litters, for example, an average 6.4 ± 2.5 pups per litter (*n* = 11 L) compared to dHet‐sired litters 5.6 ± 1.9 pups per litter (*n* = 42 L). The above data suggests that, like in *Hsd17b3* KO males, postnatal male reproductive tract development is maintained and fertility is unaffected in dKO males.

### 3.8 Testicular androgen biosynthesis is maintained in *Hsd17b3* and *Srd5a1* double knockout mice

**FIGURE 8:**
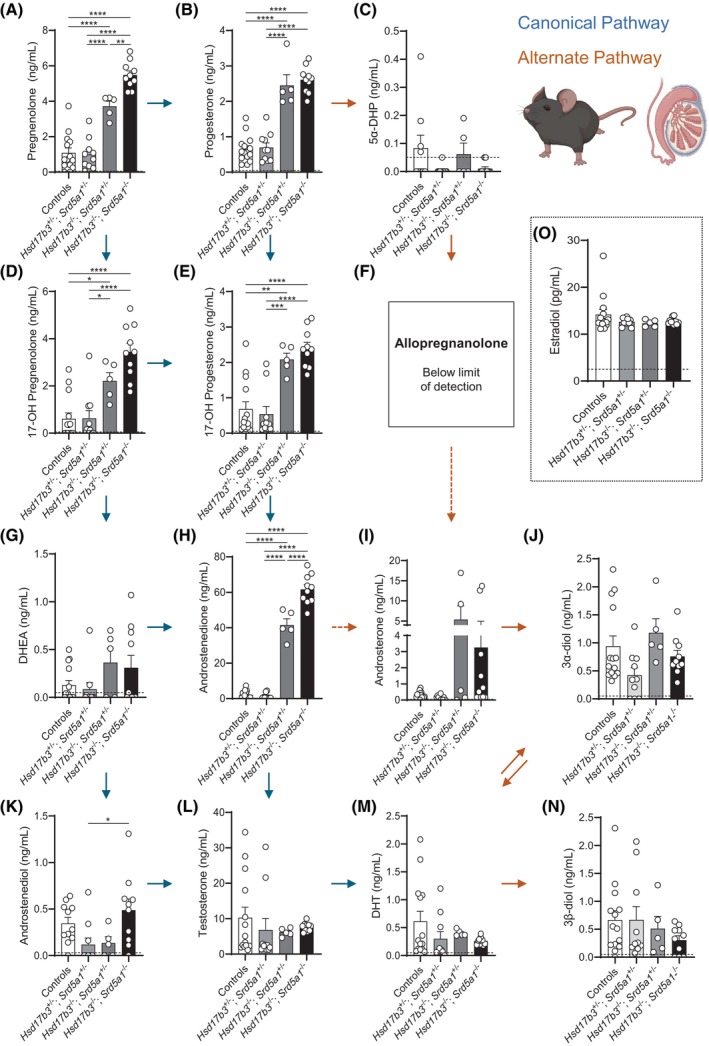
Analysis of intratesticular steroids in *Hsd17b3* ^−/−^ knockout (KO) and *Hsd17b3* ^−/−^; *Srd5a1* ^−/−^ double knockout (dKO) adult (day 80) males. (A–N) Steroid quantification of testicular androgen precursors and active androgens involved in the canonical and alternate androgen production pathways. Steroids measured include (A) pregnenolone, (B) progesterone, (C) 5α‐dihydroprogesterone (5α‐DHP), (D) 17‐OH pregnenolone, (E) 17‐OH progesterone (F) allopregnanolone, (G) dehydroepiandrosterone (DHEA), (H) androstenedione, (I) androsterone, (J) 5α‐androstane‐3α, 17β‐diol (3α‐diol), (K) androstenediol, (L) testosterone, (M) dihydrotestosterone (DHT), (N) 5α‐androstane‐3β, 17β‐diol (3β‐diol), and (O) estradiol. Blue arrows indicate the direction of the canonical androgen production pathway. Orange arrows indicate conversion occurring in the alternate androgen production pathway. Dotted arrows indicate an indirect conversion. Biological replicates that were below the limit of detection were recorded as 0 ng/mL. The limit of detection ranged from 2.5 pg/mL to 0.05 ng/mL depending on the analyte and is indicated by a dotted black line on the *y*‐axis. One‐way ANOVA, Tukey's test, where *p* ≤ .05, data shown as mean ± SEM with *n* = 5–14 per group. Significant differences between groups are indicated as *p ≤ .05, **p ≤ .01, ***p ≤ .001, ****p ≤ .0001.

Previous studies have demonstrated that testicular testosterone production is maintained in *Hsd17b3* KO mice^22^ and therefore we analyzed testicular steroids in both *Hsd17b3* KO and dKO mice. Pregnenolone, progesterone, 17‐OH pregnenolone, 17‐OH progesterone, and androstenedione were all significantly increased in the testis of *Hsd17b3* KO and dKO males (Figure 8A,B,D,E,H). Importantly, pregnenolone and androstenedione were further increased in the dKO testis compared to *Hsd17b3* KO (Figure 8A,H). This data suggests that steroid precursors accumulate when the canonical pathway is impaired in *Hsd17b3* KO and that there is a further accumulation of steroid precursors when both the canonical and alternate pathways are impaired in the testes of dKO mice.

As previously demonstrated, *Hsd17b3* KO mice continued to synthesize normal basal levels of testosterone and DHT (Figure 8L,M).^15^, ^18^ Normal intratesticular testosterone levels were also observed in dKO testes (Figure 8L) and, surprisingly, intratesticular DHT levels were also normal in the absence of SRD5A1 (Figure 8M).

Analysis of alternate pathway steroids revealed that 5α‐DHP levels were mostly below the limit of detection in the testis of all genotypes (Figure 8C) and allopregnanolone was not detectable (Figure 8F). In *Hsd17b3* KO testes, there were no significant changes in alternate pathway steroids compared to controls, suggesting that this pathway in the testis is largely unaffected by the loss of HSD17B3. Levels of the alternate pathway steroid androsterone were < 1 ng/mL in WT and dHet testes, but some *Hsd17b3* KO and dKO mice exhibited high levels (Figure 8I), perhaps consistent with increased alternate pathway activity in terms of androsterone production; however, the reason for the variation is not clear. Strikingly, alternate pathway steroids that require SRD5A, including androsterone, 3α‐diol and 3β‐diol, were not significantly altered by the loss of SRD5A1 in dKO mice (Figure 8I,J,N).

Taken together, the above data suggest that the production of DHT and other steroids in the alternate pathway of androgen synthesis in the testis is preserved in the absence of SRD5A1 in dKO mice.

Finally, to investigate whether the levels of androstenedione and testosterone were influenced by conversion into estrogens via the aromatase enzyme, testicular estrone and estradiol were measured. Intratesticular estrone levels were below the limit of detection (data not shown), and estradiol concentrations remained normal across the different genotypes (Figure 8O), suggesting that testicular aromatase activity is not altered in these mice.

### 3.9 The ablation of *Hsd17b3* and *Srd5a1* increases the expression of key steroidogenic genes in adult testes

**FIGURE 9:**
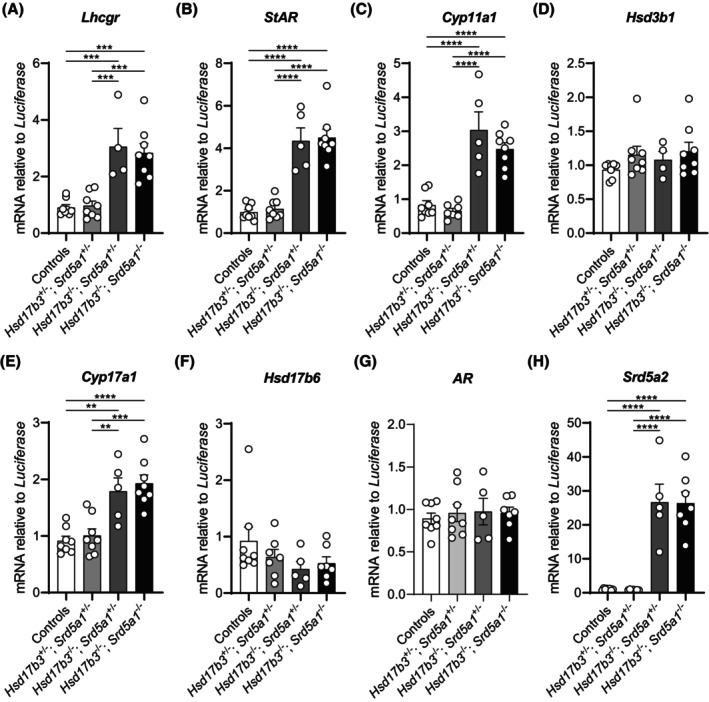
Elevated expression of key steroidogenic enzymes in the adult testes of *Hsd17b3* KO and *Hsd17b3* and *Srd5a1* double knockout (dKO) mice. mRNA transcript levels in the adult testis relative to a luciferase external control. Transcripts quantified include (A) Luteinizing hormone/choriogonadotropin receptor (*Lhcgr*), (B) steroidogenic acute regulatory protein (*StAR*), (C) cholesterol side‐chain cleavage enzyme (*Cyp11a1*), (D) 3β‐hydroxysteroid dehydrogenase type 1 (*Hsd3b1*), (E) cytochrome P450 family 17 subfamily A member (*Cyp17a1*), (F) 17β‐hydroxysteroid dehydrogenase type 6 (*Hsd17b6*), (G) androgen receptor (*AR*), and (H) steroid 5α‐reductase type 2 (*Srd5a2*). One‐way ANOVA, Tukey's test where *p* ≤ .05, data shown as mean ± SEM with *n* = 5–8 per group. Significant differences between groups are indicated as **p ≤ .01, ***p ≤ .001, ****p ≤ .0001.

The above data suggests that steroidogenesis is preserved in both *Hsd17b3* KO and *Hsd17b3* and *Srd5a1* dKO mice, and thus we next investigated the testicular expression of steroidogenic enzymes. As previously observed in mice lacking *Hsd17b3*,^20^, ^22^ the Leydig cells of both *Hsd17b3* KO and *Hsd17b3* and *Srd5a1* dKO adult mice exhibited a phenotype of increased LH responsiveness and increased production of key steroidogenic enzymes, consistent with a phenotype of steroidogenic compensation.^20^ *Hsd17b3* KO and dKO testes exhibited elevated expression of luteinizing hormone/choriogonadotropin receptor (*Lhcgr*) and steroid biosynthetic enzymes, including *StAR*, *Cyp11a1*, and *Cyp17a1* (Figure 9A–CE). Of note, *Cyp17a1* is involved in both the canonical and alternate pathways of androgen biosynthesis (Figure 1). There were no differences between genotypes in testicular expression of *Hsd3b1* (Figure 9D), as previously observed in *Hsd17b3* KO mice.^20^, ^22^ *Hsd17b6*, a steroidogenic enzyme involved in the alternate pathway, showed no changes in transcript levels between any of the groups (Figure 9F). *Hsd17b5*, another enzyme involved in the alternate androgen pathway and known to produce testosterone, was also assessed; however, transcript levels were undetectable in all genotypes.

As mRNA expression of steroidogenic enzymes was increased in *Hsd17b3* KO and dKO male mice (Figure 9A–CE), AR expression was also assessed to see if it was altered. However, testicular mRNA expression of the AR was unchanged across all genotypes (Figure 9G).

Strikingly, mRNA expression of the other steroid SRD5A enzyme, *Srd5a2*, was very low in the testes from control and dHet mice but was markedly and significantly increased >40‐fold in both *Hsd17b3* KO and dKO mouse testes (Figure 9H). This data suggests that *Srd5a2* is switched on in both *Hsd17b3* KO and dKO testes and therefore could maintain production of DHT in the adult testis to compensate for impaired androgen production in these models.

In summary, this data demonstrates that, as in *Hsd17b3* KO testes, dKO also exhibits a phenotype of steroidogenic compensation. The demonstration that testicular *Srd5a2* is switched on in both *Hsd17b3* KO and dKO mice suggests that increased SRD5A2 is a compensatory response to ablation of the canonical pathway in the testis and is likely to be responsible for continued testicular production of the potent androgen DHT.

### 3.10 The testes of both *Hsd17b3* KO and dKO mice produce basal levels of testosterone and DHT that are not responsive to hCG

The above studies were performed in mice with endogenous LHCGR activation in Leydig cells via the natural ligand, LH; however, it is known that *Hsd17b3* KO mice show reduced responsiveness to the LHCGR agonist human chorionic gonadotrophin (hCG) and are unable to increase testosterone production in response to hCG.^20^ Therefore, we also investigated testicular steroidogenesis under the conditions of maximal LHCGR stimulation using exogenous hCG. hCG treatment tests the maximal ability of the Leydig cells to produce androgens and also reduces natural variation in testosterone and the impact that alpha males may have in a cage of mice.^38^

**FIGURE 10:**
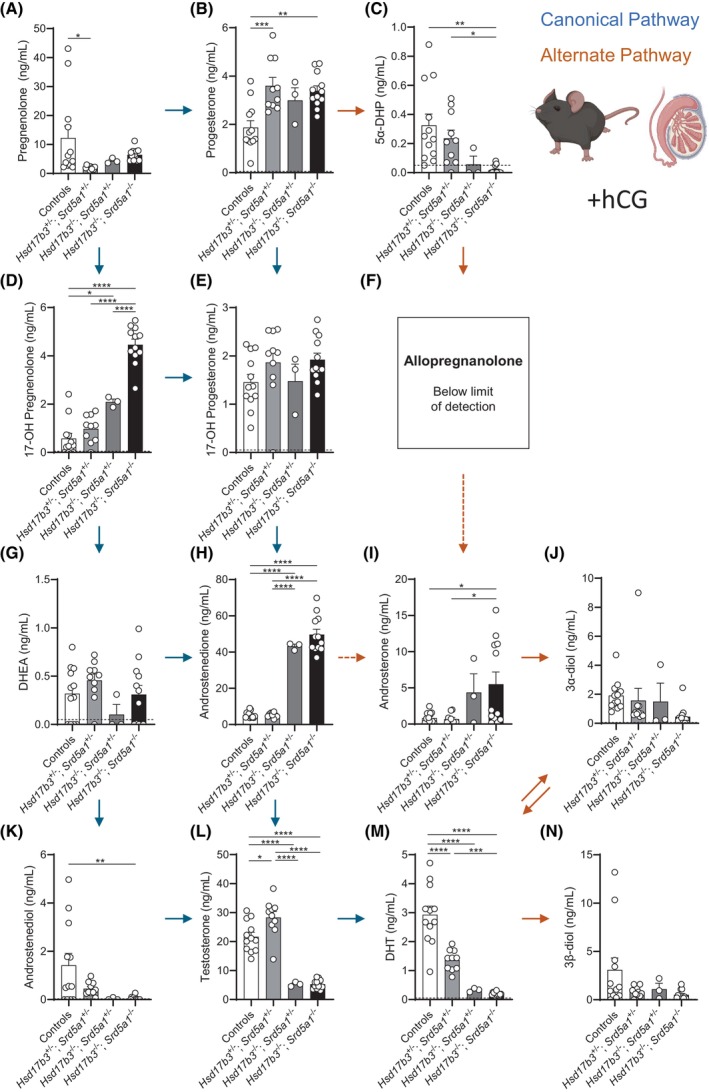
Intratesticular steroid analysis of *Hsd17b3* ^−/−^ knockout (KO) and *Hsd17b3* ^−/−^; *Srd5a1* ^−/−^ double knockout (dKO) mice following luteinizing hormone/chorionic gonadotrophin receptor (LHCGR) activation. LHCGR signaling was activated by hCG administration, and androgen precursors and active androgens in the canonical and alternate androgen production pathways were measured in the testes of day 80 adults. Steroids quantified include (A) pregnenolone, (B) progesterone, (C) 5α‐dihydroprogesterone (5α‐DHP), (D) 17‐OH pregnenolone, (E) 17‐OH progesterone, (F) allopregnanolone, (G) dehydroepiandrosterone (DHEA), (H) androstenedione, (I) androsterone, (J) 5α‐androstane‐3α, 17β‐diol (3α‐diol), (K) androstenediol, (L) testosterone, (M) dihydrotestosterone (DHT), and (N) 5α‐androstane‐3β, 17β‐diol (3β‐diol). Blue arrows indicate the direction of the canonical androgen production pathway. Orange arrows indicate the alternate androgen production pathway. Dotted arrows indicate an indirect conversion. Biological replicates that were below the limit of detection were recorded as 0 ng/mL. The limit of detection ranged from 0.01 ng/mL to 0.05 ng/mL depending on the analyte and is indicated by a dotted black line on the *y*‐axis. One‐way ANOVA, Tukey's test, where *p* ≤ .05, data shown as mean ± SEM with *n* = 3–12 per group. Significant differences between groups are indicated as *p ≤ .05, **p ≤ .01, ***p ≤ .001, ****p ≤ .0001.

After hCG stimulation, no differences were observed in canonical pathway steroids pregnenolone, 17‐OH progesterone, and DHEA between *Hsd17b3* KO or dKO mice and controls (Figure 10A,E,G). Testicular progesterone was not different between *Hsd17b3* KO mice and controls but was significantly increased in dKO mice (Figure 10B). hCG‐stimulated levels of 17‐OH pregnenolone and androstenedione were significantly increased in both *Hsd17b3* KO and dKO mice compared to controls and dHet mice (Figure 10D,H). These results are consistent with an up‐regulation of *Cyp17a1* enzyme expression in the testis (Figure 9E), as previously observed, and a reduced ability to convert androstenedione to testosterone in the absence of *Hsd17b3*.^20^, ^22^

Stimulation with hCG significantly increased testicular testosterone and DHT levels in control and dHet testes, but the levels were low in *Hsd17b3* KO and dKO mice (Figure 10L,M). This data supports previous observations in *Hsd17b3* KO mice where hCG hyperstimulation is unable to increase testosterone production, consistent with a phenotype of steroidogenic compensation, the fact that HSD17B3 is a rate‐limiting step for testosterone production, and the ability of KO Leydig cells to maintain testosterone via other, as yet unidentified, hydroxysteroid dehydrogenases.^20^

An investigation of alternate androgen pathway precursors following hCG treatment revealed that 5α‐DHP levels were significantly decreased in dKO mice compared to control and dHet mice, and the majority of 5α‐DHP measurements were below the limit of detection in dKO testes (Figure 10C). These data are consistent with a role for SRD5A1, but not SRD5A2, in 5α‐DHP synthesis in the testis (Figure 1). Intratesticular levels of androsterone were significantly increased in dKO mice compared to controls and dHet animals (Figure 10I), likely reflecting increased *Srd5a2* (Figure 9H), which can act via the alternate pathway (Figure 1); however, there were no significant differences among the genotypes in 3α‐diol and 3β‐diol (Figure 10J,N).

In summary, these data suggest that, in the absence of *Hsd17b3* and *Srd5a1*, hCG‐treated Leydig cells continue to produce abundant levels of androstenedione yet basal levels of testosterone that are likely produced by other, hitherto unidentified, hydroxysteroid dehydrogenases, similar to *Hsd17b3* KO mice.^20^ Basal production of DHT continues (Figure 8M), likely via the increased testicular expression of *Srd5a2* (Figure 9H), and thus is able to contribute to sustained androgen action in dKO testes.

### 3.11 Both *Hsd17b3* KO and dKO mice exhibit a phenotype of steroidogenic compensation

The data above details testicular steroid levels during either basal (unstimulated) conditions (Figure 8) or stimulation of Leydig cell LHCGR by hCG (Figure 10). We next investigated the hCG‐responsiveness of Leydig cell steroidogenesis in each genotype by comparing steroid levels between basal and hCG‐stimulated testis samples (Figure S4).

In control mice, hCG treatment significantly increased pregnenolone, progesterone, and 17‐OH progesterone (Figure S4A,C,D), consistent with hCG activation of Leydig cell LHCGR stimulating steroidogenesis; however, hCG stimulation of pregnenolone and 17‐OH progesterone was not observed in *Hsd17b3* KO and dKO mice (Figure 4A,D). In terms of testicular androgens, hCG treatment significantly increased testicular testosterone and DHT levels in control and dHet mice but not in *Hsd17b3* KO or dKO mice (Figure S4F,K), consistent with a loss of responsiveness to LHCGR activation, as previously demonstrated in *Hsd17b3* KO mice.^20^ Curiously, hCG stimulation in dKO mice led to a small but significant increase in 17‐OH pregnenolone and progesterone and a small but significant reduction in androstenedione compared to basal levels; however, this was not observed in *Hsd17b3* KO mice (Figure S4B,C,E), suggesting these changes could be unique to impairment of the alternate pathway.

Alternate pathway androgen precursors were also compared to determine the hCG‐responsiveness of the alternate pathway of androgen production. Both 5α‐DHP and 3β‐diol were increased by hCG in control testes (Figure S4G,J), suggesting that the alternate pathway can respond to hCG stimulation in wild‐type mice; however, the levels in *Hsd17b3* KO and dKO mice remained unchanged (Figure S4G,J), consistent with reduced responsiveness to hCG.

Overall, the data are consistent with a phenotype of compensated steroidogenesis in both *Hsd17b3* KO and dKO mice, whereby steroidogenesis is operating at a near‐maximal level of LHCGR stimulation to drive both the canonical and alternate pathways of androgen biosynthesis, and thus hCG treatment cannot induce further increases.

### 3.12 Analysis of steroids in the circulation reveals an increase in alternate pathway steroids in the absence of *Hsd17b3*, and that ablation of *Srd5a1* reduces alternate androgen biosynthesis

The above data suggests that testicular SRD5A activity is preserved in *Hsd17b3* and *Srd5a1* dKO testes, likely because of a marked up‐regulation of testicular *Srd5a2* expression. To investigate the impact of the loss of both *Hsd17b3* and *Srd5a1* on hormones in the circulation, we measured LH and steroids in the canonical and alternate pathways of androgen biosynthesis in the serum, with the latter determined using the same mass spectrometry method used to measure testicular steroidogenesis.

**FIGURE 11:**
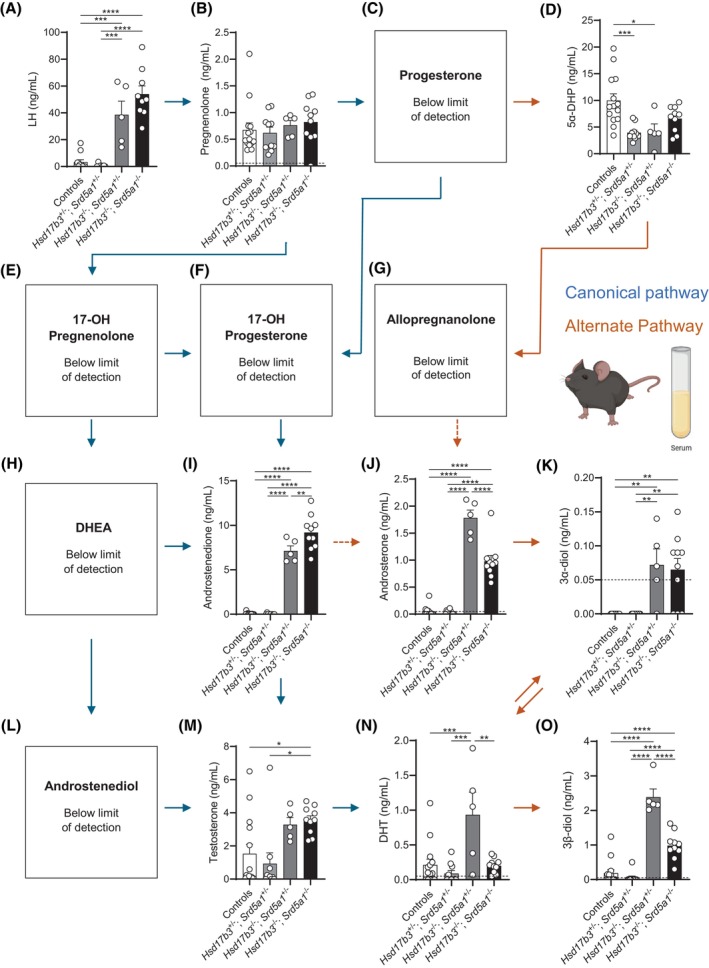
Circulating hormone analysis of adult (day 80) male *Hsd17b3* ^−/−^ knockout (KO) and *Hsd17b3* ^−/−^; *Srd5a1* ^−/−^ double knockout (dKO) mice. (A) The gonadotrophin luteinizing hormone (LH) was measured in the serum of adult mice. (B–O) Quantification of steroids involved in the canonical and alternate androgen biosynthesis pathways. Androgens and androgen precursors quantified by mass spectrometry include (B) pregnenolone, (C) progesterone, (D) 5α‐dihydroprogesterone (5α‐DHP), (E) 17‐OH pregnenolone, (F) 17‐OH progesterone (G) allopregnanolone, (H) dehydroepiandrosterone (DHEA), (I) androstenedione, (J) androsterone, (K) 5α‐androstane‐3α, 17β‐diol (3α‐diol), (L) androstenediol, (M) testosterone, (N) dihydrotestosterone (DHT), and (O) 5α‐androstane‐3β, 17β‐diol (3β‐diol). Blue arrows indicate the direction of the canonical androgen production pathway. Orange arrows indicate conversion occurring in the alternate androgen production pathway. Dotted arrows indicate an indirect conversion. Biological replicates that were below the limit of detection were recorded as 0 ng/mL. The limit of detection ranged from 0.01 ng/mL to 0.05 ng/mL depending on the analyte and is indicated by a dotted black line on the *y*‐axis. One‐way ANOVA, Tukey's test, where *p* ≤ .05, data shown as mean ± SEM with *n* = 5–14 per group. Significant differences between groups are indicated as *p ≤ .05, **p ≤ .01, ***p ≤ .001, ****p ≤ .0001.

Circulating LH was significantly increased in *Hsd17b3* KO, as previously described,^20^, ^22^ and was also increased in dKO mice compared to control and dHet males (Figure 11A). The elevated serum LH, along with increased testicular steroidogenesis, further confirms that, like *Hsd17b3* KO mice,^20^, ^22^ dKO mice show a phenotype of steroidogenic compensation, whereby mice exhibit elevated levels of LH, increased LH receptor expression (Figure 9A), and increased testicular steroidogenic enzyme expression (Figure 9B,C,E).

In terms of steroid precursors, circulating pregnenolone was not changed between genotypes (Figure 11B), and other precursor steroids prior to androstenedione formation were not detectable (Figure 11). Circulating androstenedione was significantly increased in *Hsd17b3* KO mice (Figure 11I), as observed previously.^15^, ^18^ Androstenedione levels were further increased in dKO mice compared to *Hsd17b3* KO (Figure 11I), consistent with a further accumulation of this androgen precursor when both the canonical and alternate androgen biosynthesis pathways are disrupted. No significant differences were detected in serum testosterone levels between control and *Hsd17b3* KO mice; however, there was considerable variation in the control cohort (Figure 11M), and previous studies have shown increased serum testosterone in this model.^20^, ^22^ Serum testosterone was significantly increased in dKO mice compared to controls (Figure 11M). Estrone and estradiol were not detected in the adult male circulation in all genotypes (data not shown).

In the alternate pathway, the androgen precursors androsterone, 3α‐diol, and 3β‐diol in serum were all significantly increased in *Hsd17b3* KO mice compared to controls (Figure 11J,K,O), as was serum DHT (Figure 11N). These data suggest that the loss of HSD17B3 is associated with an increase in androgen synthesis via the alternate pathway in peripheral tissues. Importantly, the loss of *Srd5a1* in dKO mice caused a marked reduction in serum androsterone, 3β‐diol, and DHT compared to *Hsd17b3* KO mice (Figure 11J,N,O). These data confirm that the ablation of *Srd5a1* in *Hsd17b3* KO mice reduces androgen biosynthesis via the alternate pathway in peripheral tissues but suggest that the alternate pathway is maintained in the testes, likely due to the compensatory increase in testicular *Srd5a2* expression.

### 3.13 Production of the androgen 11‐keto‐dihydrotestosterone (11K‐DHT) is increased in the circulation of *Hsd17b3* KO and dKO mice

The phenotypes of both *Hsd17b3* KO and dKO mice suggest the existence of multiple mechanisms of compensation when the canonical and alternate pathways of androgen biosynthesis are reduced by the ablation of the major androgen biosynthetic enzymes. Therefore, we next considered whether the production of 11‐keto androgens could be altered in these mice. This was deemed particularly relevant because observations in fetal mice with reduced testicular expression of *Hsd17b1* and *Hsd17b3* showed elevated testicular 11‐keto androstenedione and testosterone, suggesting that increased 11‐keto‐androgen production could be a compensatory response to reduced hydroxysteroid dehydrogenase activity.^27^

**FIGURE 12:**
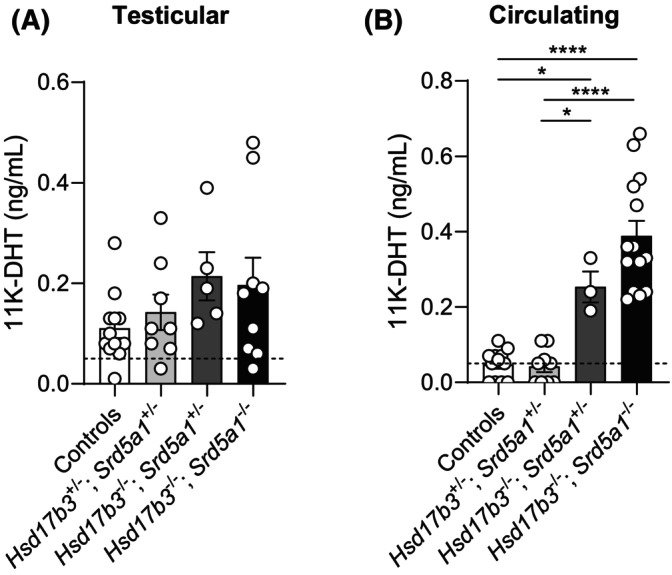
(A) Intratesticular and (B) circulating levels of 11‐keto‐dihydrotestosterone (11K‐DHT) in adult (day 80) male *Hsd17b3* ^−/−^ knockout (KO) and *Hsd17b3* ^−/−^; *Srd5a1* ^−/−^ double knockout (dKO) mice. The limit of detection was 0.05 ng/mL and is indicated by a dotted black line on the *y*‐axis. One‐way ANOVA, Tukey's test, where *p* ≤ .05, data shown as mean ± SEM with *n* = 5–14 per group. Significant differences between groups are indicated as *p ≤ .05, ****p ≤ .0001.

We therefore measured testicular and circulating levels of 11‐oxyandrogens and 11‐keto androgens in adult *Hsd17b3* KO and dKO mice. 11OH‐androstenedione, 11OH‐testosterone, 11K‐androstenedione, and 11K‐testosterone (11K‐T) were not detected in any testis or serum samples. 11K‐DHT was detectable in the testis but did not differ between genotypes (Figure 12A); however, circulating 11K‐DHT levels were significantly increased in *Hsd17b3* KO and dKO mice (Figure 12B). The concentration of 11K‐DHT in serum was similar to, and in some cases higher than, serum DHT levels (Figure 11M). These data suggest increased extra‐gonadal synthesis of 11K‐DHT when *Hsd17b3* is ablated prior to birth, suggesting that 11‐keto androgen production could be operating as another compensatory mechanism to preserve androgen bioactivity when the major androgen biosynthetic enzymes are ablated.

## 4 DISCUSSION

The canonical pathway of androgen biosynthesis involves the production of testosterone via HSD17B3 and the conversion of testosterone to DHT via the SRD5A enzymes, and the alternate pathway involves the production of DHT via SRD5A independent of testosterone production (Figure 1).^5^, ^8^, ^12^, ^14^, ^15^ Both the canonical and alternate pathways are essential for normal human male sexual development.^11^, ^12^, ^13^, ^14^, ^15^, ^36^ However, whether and how these pathways cooperate to regulate androgen bioactivity in mice is not understood. In mice, deletion of *Hsd17b3* or loss of function mutations in SRD5A enzymes do not affect male sexual development and fertility,^20^, ^22^, ^31^ suggesting compensatory mechanisms maintain androgen production when 17‐ketosteroid or SRD5A enzymes are ablated. The current study utilized mice lacking both *Hsd17b3* and *Srd5a1*, the primary SRD5A enzyme acting in the testis, to address the hypothesis that the alternate pathway of androgen biosynthesis contributes to the maintenance of male sexual development and fertility in the absence of *Hsd17b3*. The results reveal multiple mechanisms of compensation in male mice to maintain androgen bioactivity.

The impact of the alternate pathway of androgen biosynthesis in adult *Hsd17b3* KO mice was first investigated using dutasteride, a competitive inhibitor for the SRD5A1 and SRD5A2 enzymes. In *Hsd17b3* KO mice, alternate pathway precursors androsterone and 3β‐diol were significantly increased in circulation, suggesting that, when the canonical pathway is impaired, the alternate pathway is up‐regulated to preserve androgen production. The upregulation of alternate pathway precursors in *Hsd17b3* KO mice was reduced by dutasteride treatment, confirming that suppression of SRD5A reduces the entry of steroids into the alternate pathway. While dutasteride reduced circulating DHT in wild‐type mice, the levels were unaffected in *Hsd17b3* KO mice, yet seminal vesicle weights were reduced, likely reflecting a local reduction in DHT production in target tissues.^39^ No changes in testis weight or histology were seen, suggesting testicular testosterone acts directly to maintain androgen activity in the testis and/or an inability of dutasteride to compete with the high levels of testosterone for the SRD5A catalytic site. Nevertheless, this experiment revealed that alternate pathway precursors are increased in *Hsd17b3* KO mice, pointing to cross‐talk between the canonical and alternate pathways. These findings suggest that ablation of SRD5A in *Hsd17b3* KO mice may be a useful model to investigate the contribution of the alternate pathway to androgen production in the absence of HSD17B3.

Thus, we developed mice in which both the canonical and alternate androgen production pathways were impaired by deletion of *Hsd17b3* and *Srd5a1*. We deleted SRD5A1 because it is a key gateway enzyme into the alternate pathway of steroid biosynthesis (Figure 1) and the predominant SRD5A contributing to DHT production in the adult rodent testis.^29^ The phenotype of the *Hsd17b3* KO (*Hsd17b3* ^−/−^; *Srd5a1* ^*+/−*^) line generated in this study was entirely consistent with the phenotype of *Hsd17b3* KO mouse lines generated by us^20^ and others.^22^ In both *Hsd17b3* KO and *Hsd17b3* and *Srd5a1* (dKO) mice, the ablation of HSD17B3 was functionally confirmed by a decreased AGD, increased serum androstenedione levels, and a reduced ratio of testicular testosterone to androstenedione, which are hallmarks of HSD17B3 deficiency in humans^13^ and mice.^15^, ^18^ dKO mice showed a similar phenotype of steroidogenic compensation as *Hsd17b3* KO mice^20^, ^22^ and the knockout of the *Srd5a1* allele was functionally validated by significant reductions in the ratio of circulating androsterone to androstenedione and in circulating alternate androgen pathway precursors and DHT compared to *Hsd17b3* KO mice. The creation of dKO mice provides an opportunity to examine the contribution of the alternate pathway to the maintenance of androgen bioactivity in *Hsd17b3* KO mice.

The role of the alternate androgen production pathway in male prenatal sexual development was investigated by assessing dKO mice on the day of birth. Sexual development appeared normal, and steroid analyses suggested this pathway remained functional in the testes. Importantly, the expression of another SRD5A enzyme, *Srd5a2*, was significantly increased in the testes of neonatal dKO mice, suggesting it is up‐regulated to compensate for the loss of *Hsd17b3* and/or *Srd5a1*. *Srd5a2* is not up‐regulated in the absence of *Srd5a1* in female mice,^35^ suggesting that mechanisms of cooperativity may be specific to the testis. Another explanation for normal testis development in dKO mice is the maintenance of testosterone production during fetal development by continued expression of HSD17B1 that is able to compensate for the loss of HSD17B3 during fetal development.^33^ Taken together, these observations suggest that androgen action during fetal testis development in dKO mice is supported by HSD17B1 and SRD5A2.

Adult dKO mice had grossly normal reproductive tracts but decreased testicular expression of the Leydig cell maturation marker *Hsd3b6*, consistent with altered Leydig cell maturation and function during *Hsd17b3* deficiency. The data suggested that the alternate pathway operates in the adult mouse testis but that it is not up‐regulated in the testis during HSD17B3 deficiency. Intratesticular testosterone levels were preserved in both *Hsd17b3* KO and dKO, pointing to *Hsd17b3*‐independent mechanisms of testosterone synthesis.^20^ Importantly, testicular DHT was maintained in dKO mice and the mRNA expression of another SRD5A enzyme, *Srd5a2*, showed a >40‐fold increase. The data suggested that the marked up‐regulation of *Srd5a2* is a response to the loss of *Hsd17b3*, rather than the loss of *Srd5a1*. SRD5A2 is particularly effective at catalyzing 5α‐reduction at low levels of testosterone, such as is observed in the testis during puberty,^29^, ^40^ and thus it is reasonable to hypothesize that the testis responds to steroidogenic insufficiency by switching on SRD5A2. Studies in human prostate have shown that adult somatic cell suppression of SRD5A2 is regulated by DNA methyltransferase‐dependent epigenetic modifications of the SRD5A2 promoter,^41^ raising the intriguing possibility that, during *Hsd17b3* deficiency, demethylation of testicular *Srd5a2* could contribute to the increased expression of *Srd5a2*.

The testicular phenotype of dKO mice is one of steroidogenic compensation, similar to *Hsd17b3* KO mice.^20^, ^22^ dKO mice exhibited elevated circulating LH and testicular expression of *Lhcgr* and steroidogenic enzymes including *Star*, *Cyp11a1*, and *Cyp17a1* consistent with a phenotype of steroidogenic compensation by the Leydig cells.^20^, ^22^ The expression of steroidogenic enzymes was not further increased in dKO compared to *Hsd17b3* KO testes, and testicular steroids were not further stimulated by hCG, indicating that the Leydig cells are functioning to their maximum output in the absence of *Hsd17b3*. The preserved synthesis of testicular testosterone and DHT was not hCG‐responsive in dKO mice, suggesting that the compensatory mechanisms in the testis are refractory to further LHCGR stimulation. Adult testes do not express HSD17B1, even in the absence of HSD17B3,^33^ and therefore this enzyme cannot be responsible for the maintenance of testicular testosterone production in both the *Hsd17b3* KO and dKO testes, suggesting that other, as‐yet unidentified, hydroxysteroid dehydrogenase enzymes are capable of converting androstenedione to testosterone in the adult mouse testis.^5^, ^20^, ^33^

Our analyses revealed that *Hsd17b3* deficiency is associated with an increase in alternate pathway steroids and DHT in the circulation. However, this does not occur in the testes, likely due to the marked up‐regulation of testicular *Srd5a2* expression, which can maintain DHT production via the conversion from testosterone in the canonical pathway. We also showed that the loss of *Srd5a1* in the dKO mice caused a marked reduction in circulating levels of androsterone, 3β‐diol and DHT compared to *Hsd17b3* KO mice, indicating that *Srd5a1* contributes to the maintenance of androgen bioactivity in peripheral tissues in conditions of steroidogenic insufficiency. Taken together, our findings suggest that the loss of HSD17B3 in the testis and the resulting phenotype of steroidogenic compensation is associated with an increase in the alternate pathway of androgen biosynthesis in peripheral tissues. These observations point to the existence of compensatory mechanisms in extra‐gonadal tissues that act to maintain DHT biosynthesis when Leydig cell steroidogenesis is compromised.

We also assessed the levels of 11‐keto steroids in the testis and circulation. These 11‐keto steroids are synthesized from adrenal‐derived 11‐oxygenated steroids in target tissues^23^ and are present at much lower levels than native androgens in males.^23^ We were able to detect 11K‐DHT, but not 11K‐T, in the testis of adult mice, but the levels did not change in *Hsd17b3* KO or dKO mice. Circulating 11K‐T was undetectable in mice and 11K‐DHT was lowly abundant in WT mice; however, 11K‐DHT was elevated in both *Hsd17b3* KO and dKO mice, suggesting the synthesis of this bioactive androgen is an extra‐gonadal compensatory response to the loss of *Hsd17b3*. 11‐keto androgens are upregulated in castration‐resistant prostate cancer^42^ and in the fetal mouse testis when *Hsd17b1* and *Hsd17b3* expression is reduced,^27^ suggesting that they can be increased in males when steroidogenesis is compromised. 11K‐T can be efficiently converted to 11K‐DHT by SRD5A2, but not by SRD5A1,^43^ and both 11K‐T and 11K‐DHT can bind to the AR to elicit androgen responses.^28^, ^44^ Little is known about the androgenic potency of 11K‐DHT in mouse tissues, but it is important to note that androgen bioactivity relies on multiple factors, including mechanisms of local tissue production and inactivation, an ability to bind to carrier proteins and to elicit classic genomic and/or rapid non‐genomic AR‐dependent responses. The fact that 11K‐DHT, but not 11K‐T, is up‐regulated in the circulation, but not testes, of KO mice with a phenotype of androgen biosynthetic enzyme insufficiency and steroidogenic compensation points to the existence of intriguing mechanisms of co‐operativity between androgen biosynthetic pathways in mice.

Our finding that SRD5A and the alternate pathway can support androgen production in conditions of steroidogenic insufficiency is relevant to the understanding of human male endocrine disorders. Masculinization of the human fetus involves both the canonical and alternate pathways of androgen biosynthesis.^12^, ^17^ Alternate pathway androgen precursors are not produced in the developing human testis and are instead synthesized predominantly in peripheral tissues and the placenta.^14^ How the canonical and alternate pathways intersect and cooperate during human male sexual development is not clear, but our data suggest mechanisms of compensation between the two pathways to maintain androgen bioactivity. Human XY individuals with HSD17B3 deficiency can develop male characteristics during puberty^19^, ^21^; however, whether the synthesis of DHT via the alternate pathway could promote androgen‐dependent pubertal masculinization in conditions of human HSD17B3 deficiency is not known and yet is suggested by our findings in mice. Finally, ~10% of men with late‐onset hypogonadism exhibit a phenotype of compensated hypogonadism, with high LH and normal testosterone,^45^ similar to steroidogenic compensation in HSD17B3‐deficient adult mice.^20^, ^22^ Whether SRD5A and the alternate pathway, including an up‐regulation of SRD5A2 in the testis, contribute to the maintenance of androgen bioactivity in this clinical setting is unknown.

In conclusion, observations from *Hsd17b3* KO and *Hsd17b3* and *Srd5a1* dKO mice have revealed striking mechanisms of compensation to maintain androgen bioactivity during fetal life and in adulthood in male mice. In the absence of *Hsd17b3* and *Srd5a1*, fetal sexual development and androgen production can be maintained by testicular HSD17B1^33^ and there is a compensatory increase in testicular SRD5A2 expression at birth. In adult mice deficient in *Hsd17b3* alone or *Hsd17b3* and *Srd5a1*, the testes exhibit a phenotype of steroidogenic compensation, with elevated LH and precursor steroid production and continued testosterone production, likely via unknown enzyme(s) capable of synthesizing testosterone,^20^, ^22^ and DHT, likely via the marked upregulation of the SRD5A2 enzyme. In peripheral tissues, the absence of HSD17B3 causes 11K‐DHT production to be switched on, and there is an increase in steroids produced via the alternate pathway of androgen production, suggesting that the canonical, alternate, and 11‐keto steroid androgen production pathways can cooperate to contribute to androgen production. We conclude that mice have evolved multiple mechanisms, involving multiple pathways of androgen production, to maintain androgen biosynthesis throughout development and adulthood.

## AUTHOR CONTRIBUTIONS

B. M. Lawrence, D. Rebourcet, and L. B. Smith conceived and designed the research; B. M. Lawrence, D. Rebourcet, A.‐L. Gannon, S. Smith, M. K. Curley, A.‐L Darbey, and R. McKay performed the research and acquired the data; B. M. Lawrence, L. O'Donnell, P. J. O'Shaughnessy, D. Rebourcet, and L. B. Smith analyzed and interpreted the data; B. M. Lawrence, L. O'Donnell, and L. B. Smith wrote the manuscript; D. Rebourcet and P. J. O'Shaughnessy revised the manuscript.

## DISCLOSURES

The authors declare no conflict of interest.

## Supporting information


Data S1.

## Data Availability

The data that support the findings of this study are available in the Materials and Methods, Results, and/or Supplemental Material of this article. Images used in the figures were created with BioRender.com, agreement numbers NO278I5F0T, VL278I6056, PF278I6AH5, ZI278I6J27, NB278I6YC9, OK278IFGLJ, XU278IH6UG, and DI27GXZCM9.
