# Supplementary material for: Compensatory mechanisms that maintain androgen production in mice lacking key androgen biosynthetic enzymes
Source: FASEB J. 2024 Nov 18;38(22):e70177. doi: 10.1096/fj.202402093R (PMC11698012; doi:10.1096/fj.202402093R)
Supplement: Supplementary file 1 — Data S1. [file FSB2-38-e70177-s001.pdf]

Supplemental Figure 1.

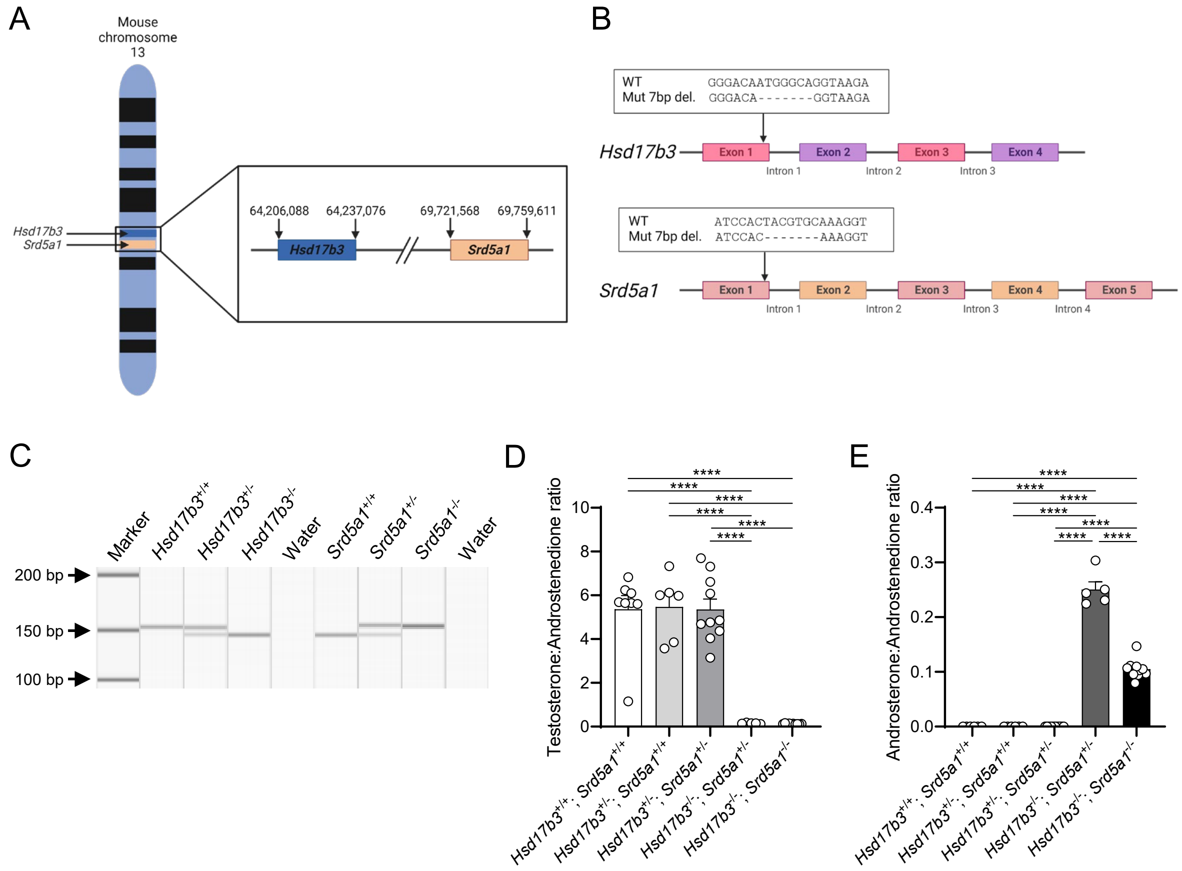

Supplemental Figure 1. Creation and validation of *Hsd17b3*$^{-/-}$; *Srd5a1*$^{-/-}$ double knockout (dKO) mice. (**A**) Schematic indicating the proximity of the genes *Hsd17b3* and *Srd5a1* on the mouse chromosome 13 indicated by the base pair numbers as per the National Center for Biotechnology Information [NCBI]. (**B**) Schematic demonstrating the 7-base pair (bp) deletions made to the *Hsd17b3* and *Srd5a1* genes to form the frameshift mutations (mut). The location of where the frameshift mutation occurred indicated by the arrow. (**C**) *Hsd17b3* and *Srd5a1* genotypes of the mice were determined using standard PCR. The presence of the *Hsd17b3*$^{+/+}$ (wild-type [WT]) gene was indicated by a 153-base pair (bp) band. *Hsd17b3*$^{-/-}$ (*Hsd17b3* KO) gene was indicated by a 7 bp deletion which showed a 146 bp band. The presence of the *Srd5a1*$^{+/+}$ (WT) gene was indicated by a 146 bp band and the *Srd5a1*$^{-/-}$ (*Srd5a1* KO) was indicated by a 153 bp band. Heterozygotes displayed both the WT and KO genes. (**D**) Adult day 80 intratesticular testosterone to androstenedione ratio for all genotypes collected. (**E**) Circulating androsterone to androstenedione ratio for all genotypes collected. One-way ANOVA, Tukey's test where $p \leq 0.05$, data shown as mean ± SEM with n=5-10 per group. Significant differences between groups are indicated as **** = $p \leq 0.0001$.

Supplemental Figure 2.

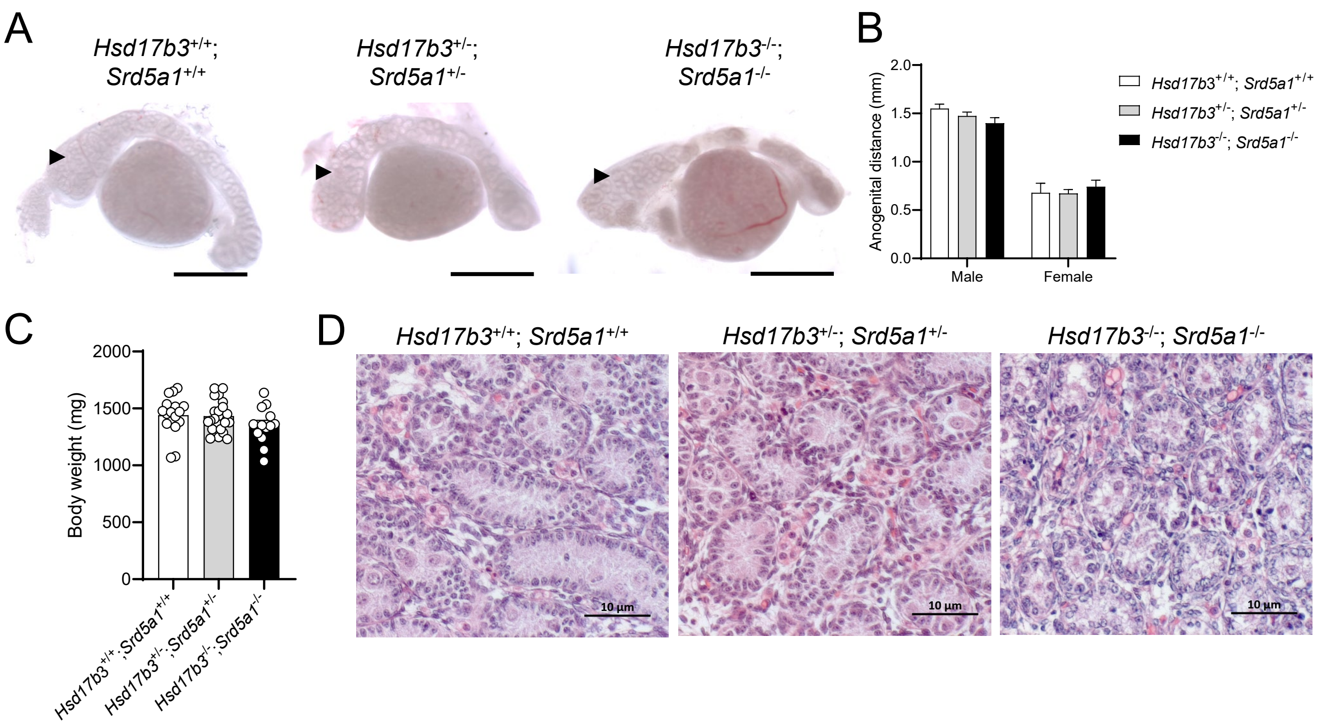

Supplemental Figure 2. Disruption to both canonical and alternate pathways of androgen biosynthesis did not impact fetal reproductive tract development in male mice. (**A**) Images of the testis and epididymis of *Hsd17b3*$^{+/+}$; *Srd5a1*$^{+/+}$ (wild-type [WT]), *Hsd17b3*$^{+/-}$; *Srd5a1*$^{+/-}$ (double heterozygotes [dHet]) and *Hsd17b3*$^{-/-}$; *Srd5a1*$^{-/-}$ (double knockout [dKO]) male mice on the day of birth (day 0). Arrow indicates epididymal coiling. Scale bar: 1mm. (**B**) Anogenital distance of male and female WT, dHet and dKO mice at day 0. Two-way ANOVA, Tukey's test, data shown as mean ± SEM with males n=13-24 and females n=5-18 per group. (**C**) Body weight of WT, dHet and dKO male mice at day 0. One-way ANOVA, Tukey's test, data shown as mean ± SEM with n=13-25 per group. (**D**) Hematoxylin and eosin staining of day 0 WT, dHet and dKO testis. Scale bar: 10μm.

Supplemental Figure 3.

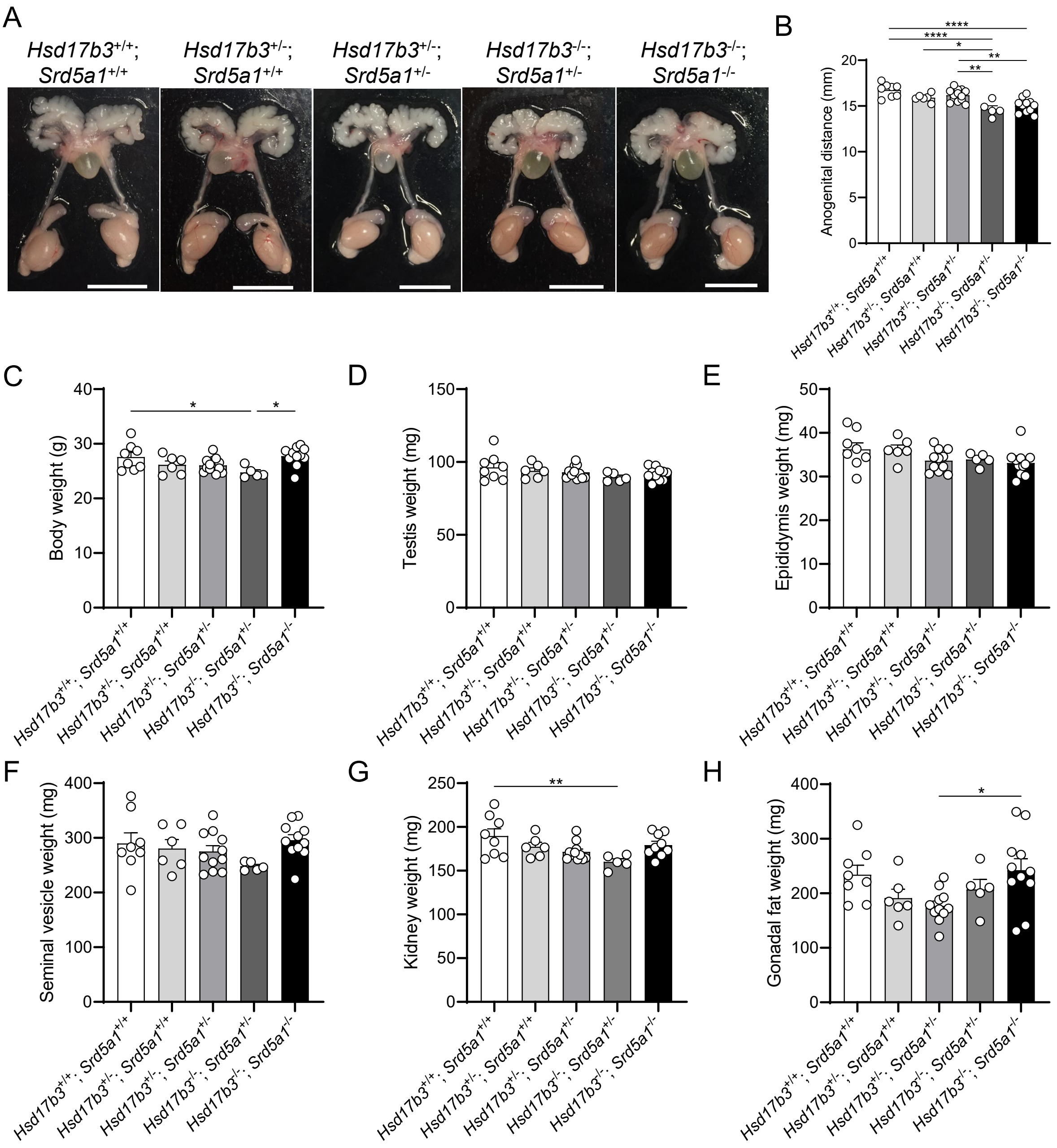

Supplemental Figure 3. Disruption to canonical and alternate androgen production pathways does not impact postnatal male sexual development. (**A**) Representative images of the day 80 adult male reproductive tract in *Hsd17b3*$^{+/+}$; *Srd5a1*$^{+/+}$ (wild-type [WT]), *Hsd17b3*$^{+/-}$; *Srd5a1*$^{+/+}$ (*Hsd17b3* Het, *Srd5a1* WT), *Hsd17b3*$^{+/-}$; *Srd5a1*$^{+/-}$ (double heterozygous [dHet]), *Hsd17b3*$^{-/-}$; *Srd5a1*$^{+/-}$ (*Hsd17b3* KO) and *Hsd17b3*$^{-/-}$; *Srd5a1*$^{-/-}$ (double knockout [dKO]) mice. (**B**) Anogenital distance and (**C**) body weight of all collected genotypes. (**D**) Weights of androgen-dependent tissues including the testis, (**E**) epididymis, (**F**) seminal vesicle, (**G**) kidney and (**H**) gonadal fat. One-way ANOVA, Tukey's test, where $p \leq 0.05$, data shown as mean ± SEM with n=5-11 per group. Significant differences between groups are indicated as * = $p \leq 0.05$, ** = $p \leq 0.01$, **** = $p \leq 0.0001$.

Supplemental Figure 4.

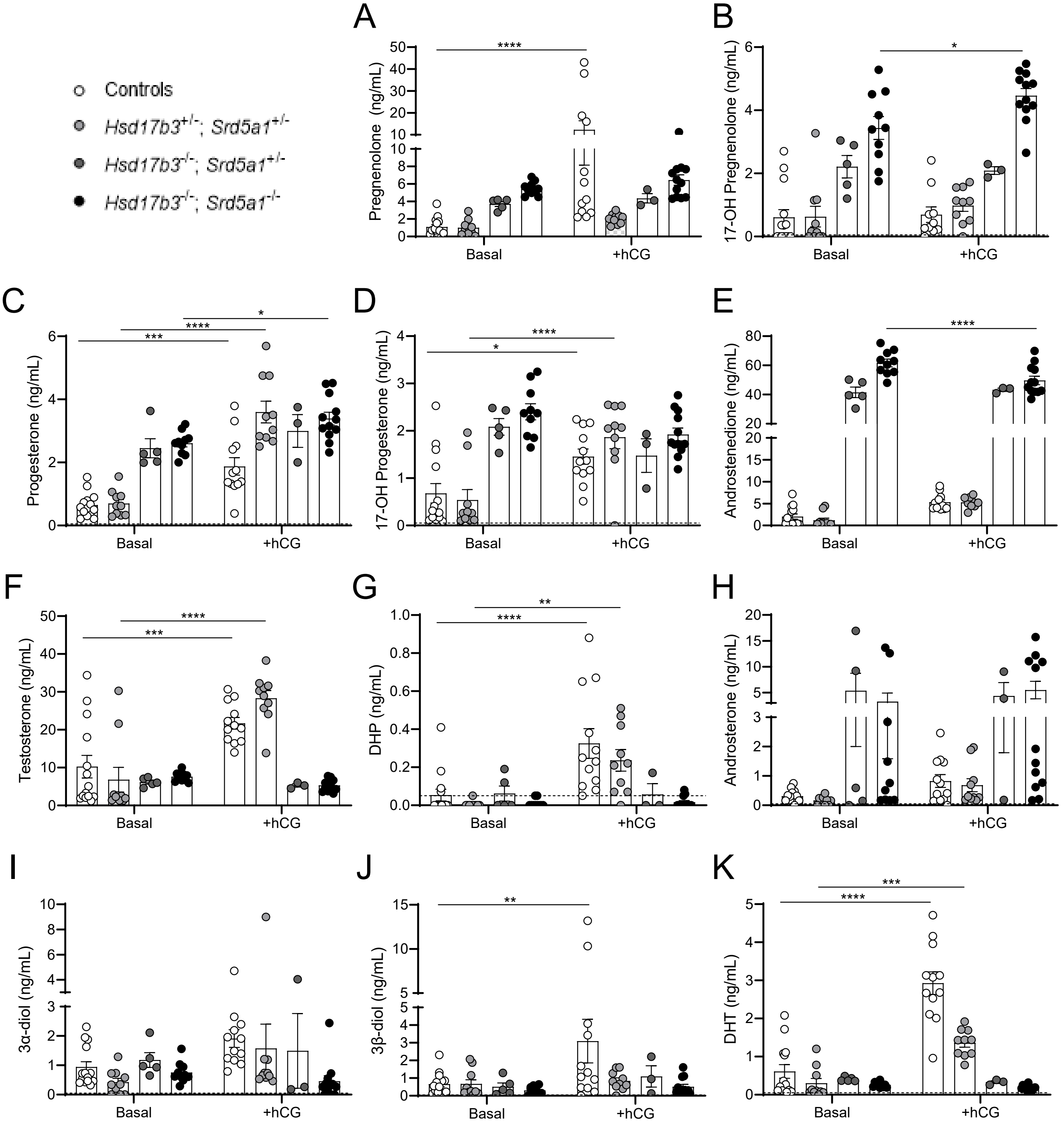

Supplemental Figure 4. Comparative analysis of basal intratesticular steroids compared with intratesticular steroids from mice treated with human chorionic gonadotrophin (hCG) to investigate hCG-responsiveness in mice with disrupted androgen production pathways. (**A-K**) Comparison of androgen precursors and active androgens in adult mice (day 80) involved in the canonical and alternate androgen biosynthesis pathways in either basal or hCG stimulated conditions. (**A**) Steroids analysed include pregenenolone, (**B**) 17-OH pregnenolone, (**C**) progesterone, (**D**) 17-OH progesterone, (**E**) androstenedione, (**F**) testosterone, (**G**) 5α-dihydroprogesterone (5α-DHP), (**H**) androsterone, (**I**) 5α-androstane-3α, 17β-diol (3α-diol), (**J**) 5α-androstane-3β, 17β-diol (3β-diol) and (**K**) dihydrotestosterone (DHT). Biological replicates that were below the limit of detection were recorded as 0ng/mL. Limit of detection ranged from 0.01ng/mL – 0.05ng/mL depending on the analyte and is indicated by dotted black line on *y*-axis. Two-way ANOVA, Sidak's multiple comparison test between basal vs hCG groups, where $p \leq 0.05$, data shown as mean ± SEM with n=3-14 per group. Significant differences between groups are indicated as * = $p \leq 0.05$, ** = $p \leq 0.01$, *** = $p \leq 0.001$, **** = $p \leq 0.0001$.

Supplemental Tables

Supplemental Table 1. Details of genotyping assays.

Assay	Reagent	Volume per reaction (µL)	Annealing Temperature (ºC)	Product size (bp)
Hsd17b3	Type-it Mastermix (2x)	5	55	WT – 150
	Hsd17b3 forward primer (20µM)	0.1		
	Hsd17b3 WT-reverse primer (20µM)	0.1		Heterozygous – 144 and 150
	Hsd17b3 del-reverse primer (20µM)	0.1		
	dH_20	3.7		Homozygous – 144
	gDNA	1		
Srd5a1	Type-it Mastermix (2x)	5	55	WT – 143
	Srd5a1 WT-forward primer (20µM)	0.1		
	Srd5a1 del-forward primer (20µM)	0.1		Heterozygous – 143 and 153
	Srd5a1 reverse primer (20µM)	0.1		
	dH_20	3.7		Homozygous – 153
	gDNA	1		

Supplemental Table 2. Primers used for genotyping assays.

Gene	Forward Primer(s)	Reverse Primer(s)
Hsd17b3	*Hsd17b3* forward: ggagaagctcttcattgctg	*Hsd17b3* WT-reverse: cttacctgcccattgtcccat
		Hsd17b3 del-reverse: cttacctgtcccattgatcg
Srd5a1	*Srd5a1* WT-forward: ctacgtgcaaaggtaatggtc	*Srd5a1* reverse: cacgaacttccaccgcttctgt
	Srd5a1 del-forward: gctatgtttctgatccacaaagg	

Supplemental Table 3. Reagents used for qRT-PCR.

**Reagent**	**Concentration Required**	**Volume per triplicate reaction (µL)**
2x Luna Universal Probe qPCR Master Mix	1x	25
Forward primer	0.4µM	1
Reverse primer	0.4µM	1
UPL Probe	0.2µM	0.6
Nuclease free water	N/A	17.4
Template DNA	0.5ng/µL	5

Supplemental Table 4. Primers and probes used for qRT-PCR assays.

Gene	Forward Primer	Reverse Primer	UPL Probe
Lhcgr	gggacgacgctaatctcg	cctggaaggtgccactgt	80
StAR	aaactcacttggctgctcagta	tgcgataggacctggttgat	83
Cyp11a1	aaggtacaggagatgctgcg	accatcttggccatgtctcc	71
Hsd3b1	gaactgcaggaggtcagagc	gcactgggcatccagaat	12
Hsd3b6	accatccttccacagttctagc	acagtgaccctggagatggt	95
Cyp17a1	catcccacacaaggctaaca	cagtgcccagagattgatga	67
Hsd17b1	ccccacggtagtgctcatt	ccgcaatgtggcataaact	82
Hsd17b5	tctcagttggtgggctgag	gcataggtaccaaaccccagt	84
Hsd17b6	cgaggagctgaggaacaaga	cccagagtcctctgtctcca	19
Srd5a2	ggtcatctacaggatcccaca	tcaataatctcgcccaggaa	50
AR	tgttatgaagcagggatgactct	ctgccagcattggagttttc	69
Luciferase	gcacatatcgaggtgaacatcac	gccaaccgaacggacattt	Custom TaqMan Probe tacgcggaatacttc

Supplemental Table 5. Steroids measured in serum and testis.

	Steroid	Type of steroid	Pathway	Limit of detection (LOD)
**1**	Pregnenolone	Androgen	Canonical	0.05ng/mL
**2**	17-OH Pregnenolone	Androgen	Canonical	0.05ng/mL
**3**	Progesterone	Androgen	Canonical [Δ4]	0.05ng/mL
**4**	17-OH Progesterone	Androgen	Canonical [Δ4]	0.05ng/mL
**5**	Androstenedione	Androgen	Canonical	0.03ng/mL
**6**	Testosterone	Androgen	Canonical	0.01ng/mL
**7**	Dehydroepiandrosterone (DHEA)	Androgen	Canonical [Δ5]	0.02ng/mL
**8**	Androstenediol	Androgen	Canonical [Δ5]	0.03ng/mL
**9**	Androsterone	Androgen	Alternate	0.05ng/mL
**10**	Androstanediol (3α-diol)	Androgen	Alternate	0.05ng/mL
**11**	Androstanediol (3β-diol)	Androgen	Alternate	0.05ng/mL
**12**	Dihydrotestosterone (DHT)	Androgen	Canonical/Alternate	0.05ng/mL
**13**	Dihydroprogesterone (5α-DHP)	Androgen	Alternate	0.05ng/mL
**14**	Allopregnanolone	Androgen	Alternate	0.05ng/mL
**15**	Estrone	Estrogen	N/A	2.5pg/mL
**16**	Estradiol	Estrogen	N/A	2.5pg/mL
**17**	Corticosterone	Glucocorticoid	N/A	0.1ng/mL
**18**	Cortisol	Glucocorticoid	N/A	0.25ng/mL
**19**	11β-Hydroxyandrostenedione (11-OH Androstenedione)	11-Oxygenated Androgen	N/A	0.02ng/mL
**20**	11β-Hydroxytestosterone (11-OH Testosterone)	11-Oxygenated Androgen	N/A	0.02ng/mL
**21**	11K-Androstenedione	Keto-androgen	N/A	0.02ng/mL
**22**	11K-Testosterone	Keto-androgen	N/A	0.02ng/mL
**23**	11K-DHT	Keto-androgen	N/A	0.05ng/mL
